# The DIAMONDS intervention for type 2 diabetes for people with severe mental illness: findings from a single-group feasibility study

**DOI:** 10.3389/frhs.2025.1688787

**Published:** 2025-11-26

**Authors:** J. V. E. Brown, C. Carswell, D. Podmore, I. Featherstone, S. Alderson, J. R. Böhnke, T. Doran, M. Hadjiconstantinou, C. E. Hewitt, R. I. G. Holt, R. Jacobs, V. Johnson, I. Kellar, J. Li, D. P. Osborn, G. Russell, J. Watson, N. Siddiqi, P. A. Coventry, the DIAMONDS Research Team

**Affiliations:** 1 Department of Health Sciences, University of York, York, United Kingdom; 2 Leeds Institute of Health Sciences, University of Leeds, Leeds, United Kingdom; 3 School of Health Sciences, University of Dundee, Dundee, United Kingdom; 4 Diabetes Research Centre, College of Life Sciences, University of Leicester, Leicester, United Kingdom; 5 NIHR Leicester Biomedical Research Centre, University of Leicester, Leicester, United Kingdom; 6 Human Development and Health, Faculty of Medicine, University of Southampton, Southampton, United Kingdom; 7 National Institute for Health Research Biomedical Research Centre, University Hospital Southampton NHS Foundation Trust, Southampton, United Kingdom; 8 Centre for Health Economics, University of York, York, United Kingdom; 9 Leicester Diabetes Centre, University Hospitals of Leicester NHS Trust, Leicester, United Kingdom; 10 School of Psychology, University of Sheffield, Sheffield, United Kingdom; 11 Division of Psychiatry, University College London, London, United Kingdom; 12 North London NHS Foundation Trust, London, United Kingdom; 13 Bradford District Care NHS Foundation Trust, Bradford, United Kingdom; 14 Hull York Medical School, York, United Kingdom; 15 School of Nursing and Public Health, Manchester Metropolitan University, Manchester, United Kingdom

**Keywords:** serious mental illness (SMI), type 2 diabetes, self-management, feasibility and acceptability, integrated care

## Abstract

Diabetes self-management is critical for improving health outcomes, but people with severe mental illness (SMI) face additional barriers that complicate effective engagement with self-management behaviours and with existing diabetes services. This feasibility study assessed the acceptability and feasibility of the DIAMONDS intervention, a tailored type 2 diabetes (T2D) self-management programme designed for people with SMI and delivered by trained coaches over 16 weekly sessions, in preparation for a future randomised controlled trial (RCT). Thirty participants with both T2D and SMI were recruited, and 29 were included in the study. The thresholds for participant recruitment and retention for progression to the RCT were met. Twenty-three participants (66%) attended at least one intervention session. Consistent weekly participation proved challenging, with only 15 participants (52%) attending eight or more (50%+) sessions. However, the intervention was acceptable to both participants and coaches, as indicated by coach session logs. High completion rates were observed for self-reported measures, while physical health data and data from primary care records had some omissions, prompting refinements in data collection for the RCT. This study highlights the feasibility and acceptability of delivering an evaluation of a structured diabetes self-management intervention in people with SMI. Some modifications to study processes will be required before moving to the main RCT, including adjustments to intervention delivery (including more flexibility in the timing of intervention sessions and coach training to improve confidence in supporting the use of a mobile app), data collection processes, and intervention fidelity assessment for the RCT, to enhance adherence and accommodating the complex needs of this population. This study represents an important step towards the development and robust evaluation of a self-management intervention to improve diabetes outcomes for people with SMI, addressing a significant gap in health equity.

**Clinical Trial Registration:**

https://www.isrctn.com/ISRCTN15328700, ISRCTN 15328700.

## Introduction

People with severe and enduring mental illness (SMI), including schizophrenia, schizoaffective disorder, bipolar disorder, and psychosis (1), face profound health inequalities that lead to a reduction in life expectancy of 10–15 years compared with the general population (2). Reducing this ‘mortality gap’, mainly attributed to co-existing physical long-term conditions, is a priority for researchers, policymakers, and service users (3–5).

Type 2 diabetes (T2D) is two to three times more common in people with SMI than in the general population and is associated with poorer outcomes (6–9). Appropriate self-management is central to improving clinical, behavioural, and psychological outcomes in people with diabetes. National and international guidelines recommend diabetes self-management education and support (DSMES) programmes, which target healthy diet, exercise, smoking cessation, self-monitoring, and medication (10, 11). However, existing DSMES programmes fail to address the specific support needs of people with SMI (12, 13). Therefore, in partnership with people with lived experience of SMI and T2D (service users and carers) and healthcare professionals (HCPs), we co-developed DIAMONDS, a DSMES intervention specifically for this population. The development process of the DIAMONDS intervention is described by Carswell et al. (14).

In line with the Medical Research Council (MRC) framework for developing and evaluating complex interventions (15), intervention components and study processes must be tested in a feasibility study before progressing to a definitive evaluation. In our case, this included intervention acceptability to participants with SMI, their carers, and the HCPs delivering the intervention and the feasibility of intervention delivery. It was also important to test processes related to recruitment and retention of participants with SMI and T2D (16, 17) and identify uncertainties related to data collection, such as the acceptability of blood tests to this population (18). The results of this feasibility study informed the protocol for a definitive RCT to evaluate the clinical and cost-effectiveness of the DIAMONDS intervention.

Specifically, the objectives of the DIAMONDS feasibility study were as follows:

- Test the feasibility of procedures for recruitment and retention of participants.
- Test the feasibility of quantitative and qualitative data collection methods.
- Undertake an exploratory economic evaluation.
- Evaluate the acceptability and feasibility of the DIAMONDS intervention.
- Develop an intervention fidelity (IF) framework for use in the DIAMONDS RCT.

## Materials and methods

This single-group feasibility study [ISRCTN registration: 15328700 (12/03/2021), https://www.isrctn.com/ISRCTN15328700] is reported in line with the CONSORT checklist for pilot and feasibility trials. Items specific to randomisation and allocation concealment are excluded, as recommended in the guidelines for reporting non-randomised pilot and feasibility studies (19, 20) (Supplementary Appendix 1). Table 1 provides an overview of the qualitative and quantitative methods used to address the study objectives. This paper reports only the quantitative methods and results for study objectives 1–5. The qualitative findings will be reported in a separate paper.

**Table 1:** Feasibility study objectives and data collection methods reported in this paper.

Study objective	Method of data collection
Test the feasibility of procedures for recruitment and retention of participants	Assessment of recruitment and participant retention, intervention, and study withdrawal using confirmed numbers of participants entering and leaving the study
Test the feasibility of quantitative collection methods	Assessment of completion rates of baseline and outcomes data, including data from primary care records, questionnaires, physical health measures (weight, height, waist circumference), HbA_1c_ blood results, and accelerometers (subset of participants)
Undertake an exploratory economic evaluation	Participant-reported health resource use collected using questionnaires Health resource use information extracted from primary care records
Evaluate the acceptability and feasibility of the DIAMONDS intervention	Assessment of participant retention rate as a proxy measure of intervention acceptability Interrogation of intervention session logs for assessment of session frequency, duration, and content
Develop an intervention fidelity framework for use in the DIAMONDS RCT	Refining existing frameworks used with comparable interventions to assess the fidelity of delivery of DIAMONDS intervention sessions Piloting of the DIAMONDS intervention fidelity frameworks by trained assessors using session recordings

Ethics approval was granted by the Research Ethics Committee Leeds West (reference: 21/YH/0059). Study oversight and governance structures are summarised in Supplementary Appendix 2.

The study methods are reported in detail in the published study protocol (21). Supplementary Appendix 3 provides an overview of amendments to the study protocol.

### Study population and setting

Participants with SMI and T2D were recruited from six secondary care National Health Service (NHS) mental health trusts in the North of England.

Table 2 summarises the eligibility criteria for participants with SMI. Eligibility was confirmed by members of the Research and Development (R&D) teams at the participating NHS Trust sites.

**Table 2:** Eligibility criteria for participants with SMI.

Criteria	Eligible	Ineligible
Age	18 years and over	Under 18 years
Setting	Community-dwelling, i.e. living independently or with family, in a supported housing scheme, or a residential care setting	Inpatients in a mental or physical health care facility
Mental health diagnosis	Clinician confirmed diagnosis of one or more of the following: Schizophrenia Schizoaffective disorder Bipolar disorder Psychosis	Any other mental health diagnosis, regardless of severity, duration, or treatment. NB: individuals who had other mental health diagnoses in addition to one of the eligible conditions were eligible for inclusion
Diabetes diagnosis	Clinician confirmed diagnosis of type 2 diabetes (insulin-treated or non-insulin-treated) of at least 3 months duration. Eligibility was not restricted by a minimum HbA_1c_ level	Any other type of diabetes, including type 1 diabetes, diabetes occurring only during pregnancy (‘gestational diabetes’), diabetes due to a specific genetic defect or secondary to pancreatitis or endocrine conditions
Cognitive function	No cognitive impairment, or mild cognitive impairment that does not hinder giving informed consent or full participation in the study	Cognitive impairment of a severity deemed to affect the person's ability to give informed consent or fully participate in the study
Capacity	Only people with the capacity to provide informed consent (as per the 2005 Mental Capacity Act) were eligible for participation	No capacity to give informed consent as per the 2005 Mental Capacity Act

### Sample size

This study took place during the Covid-19 pandemic; it was therefore necessary to balance the requirement for robust feasibility testing against the resources and capacity of our partner NHS Trusts to deliver this research. As no inferential statistics were planned for this study, we did not conduct a power calculation to determine the target sample size. Instead, we reached a consensus with the study steering committee and the funder that a target sample size of 30 would provide sufficient information to inform our planned RCT without placing undue pressure on NHS partners. This sample size was deemed sufficient to collect feasibility data on the specified outcomes and in line with the median sample size used in feasibility studies funded by the National Institute for Health and Care Research (NIHR) (22). We were confident about the feasibility of the proposed randomisation procedures from our previous experience of conducting RCTs in this population and therefore deemed a single-group feasibility study appropriate (18, 23, 24).

### Recruitment and consent procedures

Our recruitment and consent procedures were based on successful approaches from previous trials with people with SMI (18, 23, 24) and co-designed with our service-user and carer group, DIAMONDS Voice. We adopted a flexible approach to identifying potential participants with SMI, including methods such as (i) database and caseload screening; (ii) liaising with consultants, clinical teams, pharmacies, and supported housing managers; and (iii) patient self-referral. Potentially eligible people with SMI and T2D were approached about participating in the study in two stages: they were provided with a brief information sheet, and permission to contact them was confirmed, followed by a longer discussion about the study and the provision of a detailed participant information sheet. Procedures for written and verbal telephone consent were in place. R&D staff at participating NHS Trusts involved in recruitment were trained to assess participants' capacity to consent. A sample consent form and information sheet are included in Supplementary Appendix 4.

Supplementary Appendix 5 is the job description for DIAMONDS Coaches. We approached mid-level healthcare workers (NHS Agenda for Change Band 4) with either experience in working with people with SMI or experience delivering diabetes education or other behaviour change programmes. Full training was provided by colleagues at Leicester Diabetes Centre who were involved in the development of the DIAMONDS intervention. We negotiated with the employing institutions of the DIAMONDS Coaches that they would have protected time to attend the three training sessions and deliver the intervention in line with the requirements of the study. Coaches were supported with mentorship calls throughout the study.

### The DIAMONDS intervention

**Figure 1:**
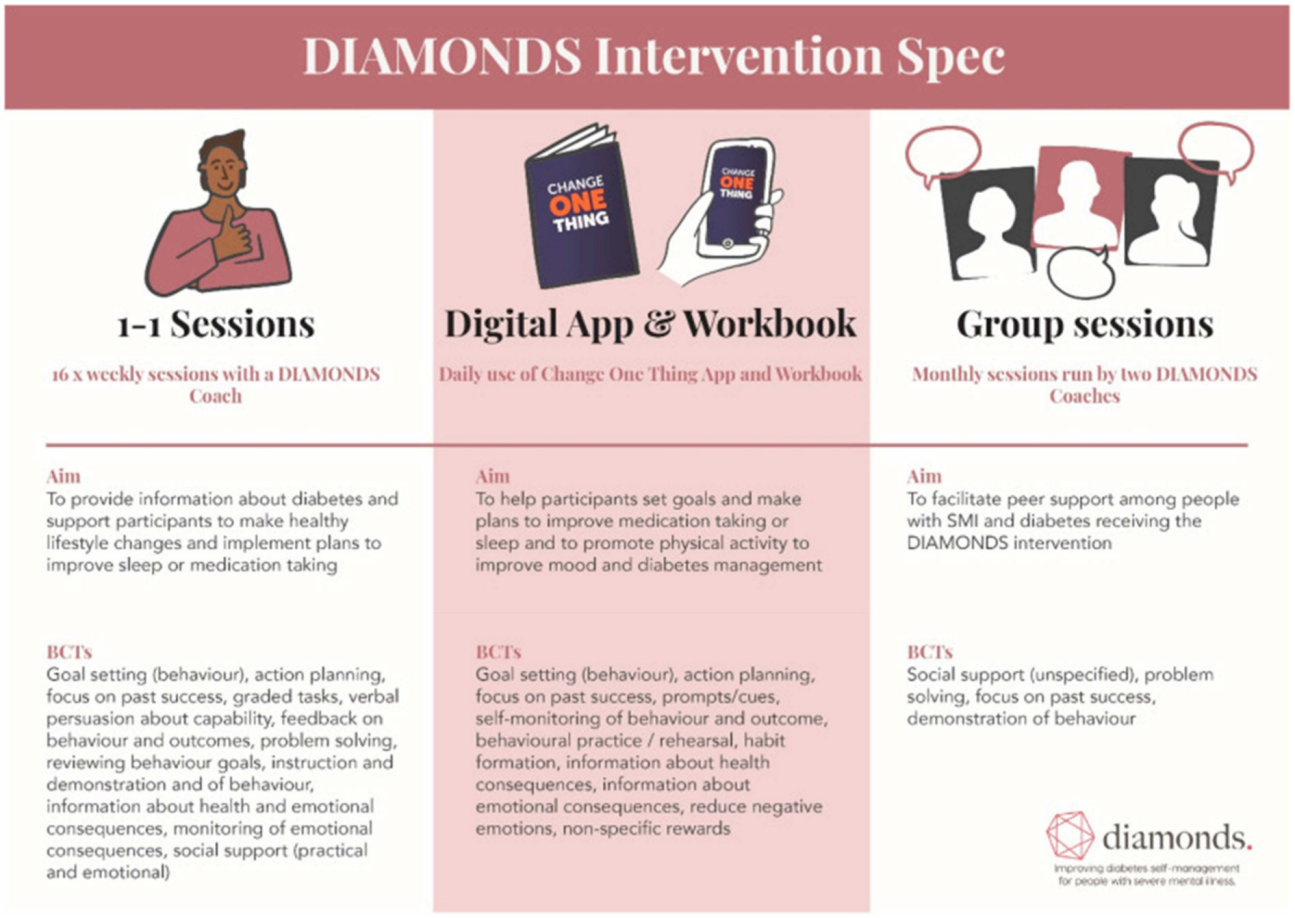
The DIAMONDS intervention (original version at the start of this feasibility study).

In this single-group feasibility study, all participants received the DIAMONDS intervention. The co-design of the intervention with people with lived experience of diabetes and SMI and HCPs is described elsewhere (14). The planned intervention involved 16 weekly one-to-one sessions between the participant and a DIAMONDS Coach, delivered over 4 months. In the sessions, the coach supported the participant to set goals and make action plans to support sustainable behaviour change with the aim of increasing diabetes understanding and management, as well as overall wellbeing. The sessions were supported by a pen-and-paper workbook and an optional mobile companion app (‘Change One Thing’). In addition, monthly peer-support group sessions were planned. Figure 1 below summarises the planned intervention, including the specific behaviour change techniques (BCTs) each component is supposed to deliver. Further detail is given in Supplementary Appendix 6.

### Data collection and analysis

#### Testing the feasibility of the study methods

We collected all planned primary and secondary outcomes for the main RCT (Table 3).

**Table 3:** Summary of data collection tested in the DIAMONDS feasibility study.

Data item	Data collection method
HbA_1c_^a^	Collected by R&D teams at recruiting sites
Sociodemographics	
* *Age, sex, ethnicity	Self-report
* *Index of multiple deprivation	Determined by the study team based on the participant's postcode
Physical health status	
* *Date diagnosed with diabetes	Primary care records
* *Height, weight, waist circumference	Measured by R&D teams at the recruiting site
* *BMI (calculated from height and weight)	Calculated by the study team based on measurements provided by R&D teams at recruiting sites
* *Blood pressure	Measured by R&D teams at recruiting sites
* *Haemoglobin^b^	Collected by R&D teams at recruiting sites
* *Lipid profile^b^	Collected by R&D teams at recruiting sites
* *Medical comorbidities	Primary care records
* *Smoking status	Self-report^c^
Mental health	
* *Type of SMI	Primary care records
* *Date diagnosed with SMI	Primary care records
* *Brief Psychiatric Rating Scale (BPRS)	Interviewer rated by trained R&D staff at recruiting sites (scored 1–7, with 1 indicating no symptoms and 7 indicating the most severe symptoms)
* *Patient Health Questionnaire-9 (PHQ-9)	Self-report (scored 0–27, with higher scores indicating more severe depression)
Diabetes measures	
Diabetes microvascular and macrovascular complications	Medical record
Diabetes distress: Problem Areas in Diabetes (PAID 5) Scale	Self-report (scored 0–20, with higher scores suggesting greater diabetes-related emotional distress)
Summary of Diabetes Self-Care Activities (SDSCA)	Self-report (11 items scored on an 8-point Likert scale with individual item scores added up to create a total score which is converted to a percentage. A higher percentage indicates better self-care behaviours)
Physical activity	
International Physical Activity Questionnaire (IPAQ)	Self-report [scored using item scores converted to Metabolic Equivalent Task (MET) minutes with higher MET scores indicating higher levels of activity]
Physical activity	Wrist-worn accelerometer
Health economic outcomes	
Health-related quality of life (EQ-5D-5L)	Self-report (scored using the relevant UK scoring algorithm)
Health/social care resource use	Self-report Primary care records
Medication for SMI	Primary care records
Medication for diabetes	Primary care records
Process evaluation measures	
Mechanisms of Action questionnaire^d^	Self-report during intervention sessions
Engagement with the Change One Thing app^d^	User data collected within the app
Acceptability of the intervention	Qualitative interviews (outcomes reported elsewhere)

a HbA_1c_ is the primary outcome for the planned DIAMONDS RCT.

b Haemoglobin and lipid profile are secondary outcomes for the DIAMONDS RCT. It was not possible to collect these outcomes in the feasibility study. The feasibility of collecting a blood sample and sending it to a lab for analysis was assessed by measuring HbA_1c_ levels.

c Participants were supported to complete self-report measures during their data collection appointment. They had the option of completing the questionnaires themselves or working with a member of the R&D team at the recruiting site who would read out the questions and record the participants’ verbal responses.

d The content management system (CMS) for the Change One Thing app was set up to collect user data throughout the intervention delivery period while participants are engaging with the app. Information about mechanisms of action was requested from participants via the app monthly.

We recorded the number of individuals approached, completing the intervention and/or signposted to other services, and providing outcome data. Trained staff in the participating NHS Trusts administered questionnaires and conducted physical measurements, recording the data in standardised case report forms. Blood samples were taken by Trust R&D staff, sent to a central laboratory [Laboratory Medicine at Manchester University NHS Foundation Trust; United Kingdom Accreditation Service (UKAS) accreditation numbers 8650 (Haematology) and 9063 (Biochemistry)], and results were returned to the study team (University of York), using participant IDs to maintain confidentiality. Data loss due to issues with processing blood samples was recorded.

Data were summarised descriptively:

- Recruitment rate was measured as the proportion of the recruitment target (*N* = 30) achieved at 5 months after the start of recruitment.
- Missing data were measured as the proportion of missing outcome data at the end of the recruitment period (5 months from the start of recruitment) for physiological and self-reported data items.
- Intervention delivery rate was recorded as the proportion of planned sessions delivered (measured as the number of completed intervention session logs per participant within 15 weeks of the first intervention session).

Coaches were asked to complete a session log using Qualtrics XM (Provo, UT, USA) after each session. We recorded the number of individuals approached, completing the intervention and/or signposted to other services, and providing outcome data. Withdrawals, with reasons where available, were also recorded.

For continuous data, we calculated means and standard deviations (or medians and interquartile ranges). For categorical data, we used the frequency and proportion of events. To evaluate intervention uptake, we summarised the number, frequency, and duration of sessions. The feasibility of our quantitative data collection methods was assessed in relation to data attrition using proportions of missing data from questionnaires and case report forms, including medical records, and the proportion of participants who declined any of the physical measures. To assess the feasibility of data collection using accelerometers, participants at two trusts were offered these devices. We recorded the number and percentage of participants who declined to wear an accelerometer as well as the extent of missing data due to problems with the hardware, the download processes, interruptions in the wear time, or loss of devices.

#### Exploratory economic evaluation

An exploratory economic evaluation was conducted to evaluate the feasibility of collecting health and social care resource use and health-related quality of life data for the economic evaluation in the RCT. Training and intervention delivery materials and activities were recorded by the research team at the University of York. Opportunity costs for staff involvement were estimated based on their recorded wage bands, plus salary oncosts. Costs of materials and postage, and operation costs of the ‘Change One Thing’ mobile app were extracted from study records. All costs were presented in pound sterling using 2021 prices.

Two methods were used at baseline to collect data on participants' use of health (primary and secondary care) and social care resources: a self-report questionnaire and medical record review. Questionnaire response rates and rates of missing data at the item level were evaluated. The accuracy of the responses and the response consistency within the same participant were checked to ensure that the questionnaires could yield accurate and reliable results. Data on participants' use of medication for diabetes and SMI were also collected from their medical records. The EQ-5D-5L self-report questionnaire was used to measure participants' health-related quality of life.

#### The acceptability and feasibility of the DIAMONDS intervention

Quantitative methods to evaluate the acceptability and feasibility of the intervention included the following:

- The number of intervention sessions completed and the number of participants who withdrew from the intervention were used as proxy measures of intervention acceptability.
- Intervention session logs were interrogated to assess session frequency, duration, and content to evaluate the feasibility of the intervention and its delivery.
- The feasibility and acceptability of coach training were evaluated (see further details below).

#### Development of an intervention fidelity framework for use in the DIAMONDS RCT

To assess fidelity in delivering the DIAMONDS intervention, bespoke intervention fidelity (IF) tools—a checklist and coding manual—were developed by the Leicester Diabetes Centre and the University of York team. The tools were created through a four-step process: (i) reviewing existing IF measures; (ii) designing a tailored checklist and manual based on coach training materials, intervention specifications, and BCTs; (iii) refining tools with feedback from DIAMONDS researchers, trainers, and developers; and (iv) piloting the tools with experienced coders to evaluate inter-rater reliability (25).

**Figure 2:**
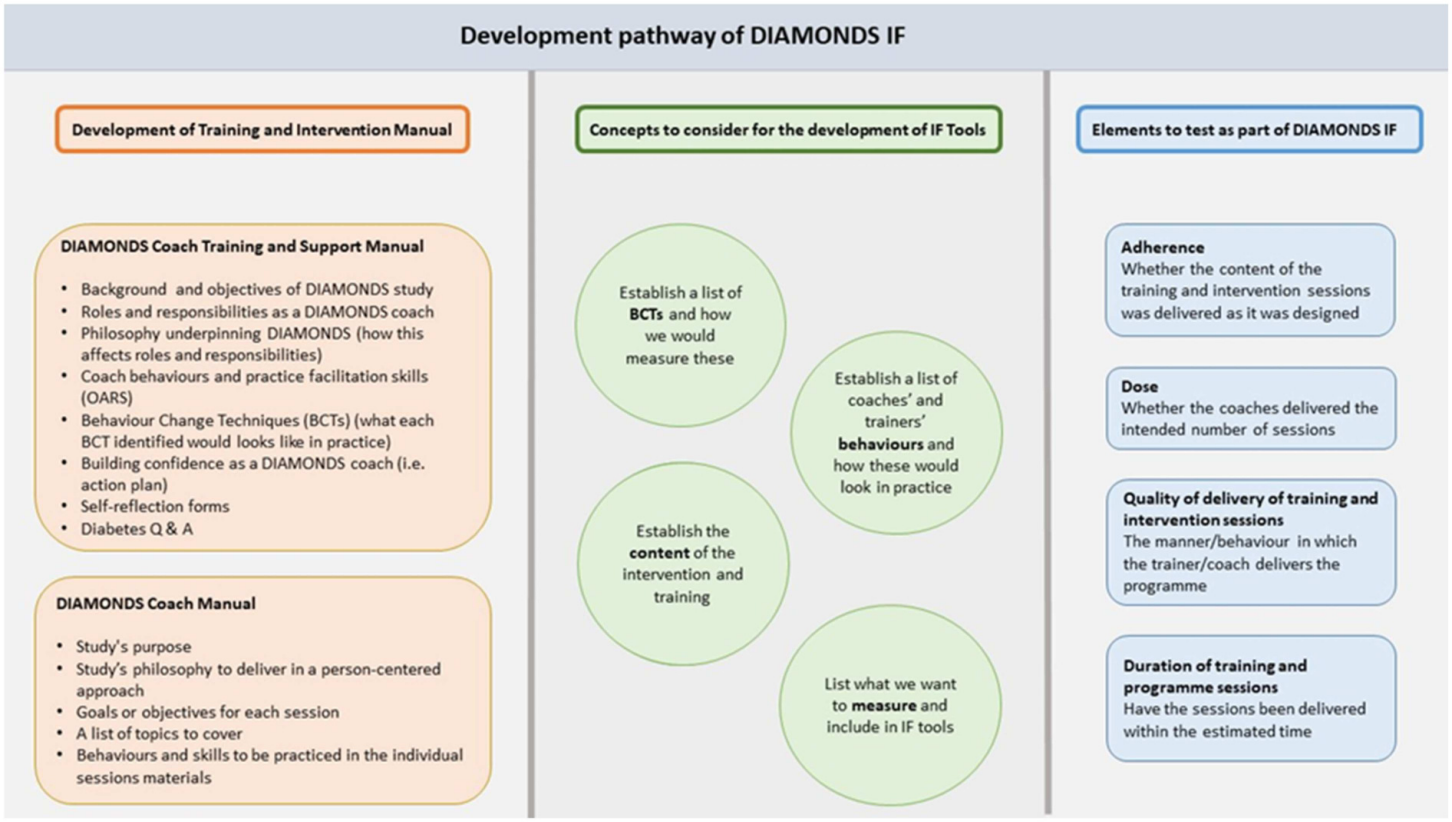
Development pathway of the DIAMONDS intervention fidelity (IF) assessment tools.

These tools, specific to DIAMONDS, enabled measurement of key fidelity dimensions (Figure 2):

- Adherence: whether session content and BCTs were delivered as intended
- Exposure and dose: number and duration of sessions
- Quality of delivery: how coaches delivered sessions, including use of BCTs
- Duration: whether sessions adhered to expected timing

This process supported the development of robust, programme-specific fidelity measures.

Intervention fidelity (IF) was assessed using audio recordings of one-to-one sessions. Coaches were asked to record one first, one core (sessions 2–15), and one final session per consenting participant, aiming to sample up to 10% of all sessions. Recordings were made with encrypted devices. Two of three trained coders at the Leicester Diabetes Centre independently used the IF checklist and coding manual to rate each session's content, duration, behaviours, and BCTs as ‘Present’, ‘Absent’, or ‘Attempted’. Discrepancies were discussed and, if unresolved, a third coder made the final decision. Tools were refined iteratively after each coding round.

#### Evaluation of DIAMONDS coach training

Evaluation of the DIAMONDS coach training was undertaken using pre- and post-training questionnaires, which included coaches' receipt of the training content as well as satisfaction and confidence with their role. This evaluation, informal feedback from researchers, and data from the IF recordings were used to iteratively refine the training plan between cohorts of coaches trained. Evaluation forms were updated where necessary to reflect these changes.

### Service-user and carer involvement

DIAMONDS Voice, our group of people with lived experience of SMI and physical health problems (service users and carers), made critical contributions to the design and conduct of this study. Specifically, they played key roles in developing participant-facing documents, refining the content and presentation of intervention materials, and shaping the proposed data collection methods, including questionnaires and physical measurements.

### Progression criteria

The progression criteria from the feasibility study to the RCT were as follows:

- The recruitment of at least 20 participants with red–amber–green stop–go ratings based on <20 participants (red); 20–25 (amber); 26–30 (green).
- At least 50% of the intervention sessions delivered to 80% of participants
- A self-management intervention that is acceptable and feasible to participants and coaches

## Results

All study objectives were met as reported below.

### Recruitment and participant characteristics

#### Feasibility of recruitment and retention

**Figure 3:**
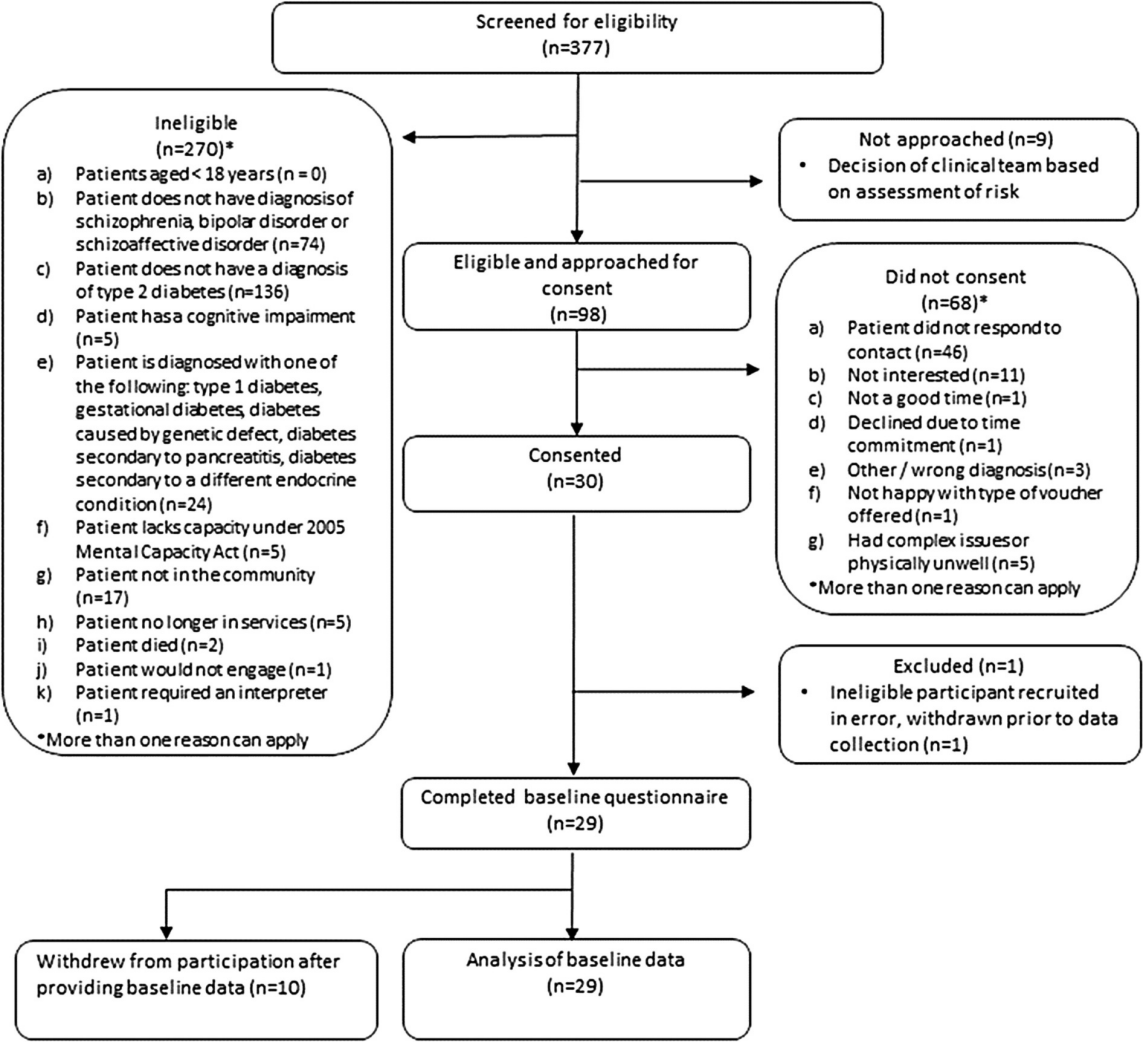
Flow diagram of screening and recruitment.

Recruitment took place from 1 July to 30 November 2021. A total of 377 people were screened for eligibility, of whom 107 (28.4%) were identified as eligible for participation. The main reasons for ineligibility were the person not having a diagnosis of T2D (*n* = 136) or no diagnosis of an eligible SMI (*n* = 74). Ninety-eight of those eligible were approached for participation, and 30 (30.6%) consented to take part. Recruitment to the feasibility study ended when the target was reached. One participant with type 1 diabetes was recruited in error and was withdrawn prior to baseline data collection (Figure 3). This occurred because the participant, recruited from a mental health service, misunderstood the status of their newly diagnosed diabetes, and the corresponding physical health record had not yet been updated to specify diabetes type. Going forward, participant self-declarations about diabetes status will be verified against general practice (GP) or other health records prior to formal recruitment. The progression criterion was in ‘green’ for recruitment (26–30 participants).

#### Participant characteristics

The mean age of participants was 52.3 years (SD 8.3), 16 (55%) were men, 20 (69%) were White British (*n* = 20, 69%), and the majority had obesity (*n* = 24, 88.9%).

#### Data collection completion rates for participant demographic data

Self-reported measures yielded the most complete data, with 100% of participants providing age, gender, and ethnicity data (Table 4). Data for the physical measures taken by the R&D teams at the recruiting sites were incomplete: BMI (93.1% complete), blood pressure (86.2%), and waist circumference (72.4%).

**Table 4:** Participant characteristics and data collection completion rates.

Characteristic	Participants analysed (*n* = 29)
Age (years)	
Number (%) with data	29 (100)
Mean (SD)	52.3 (8.3)
Gender, *n* (%)	
Number (%) with data	29 (100)
Female	13 (44.8)
Male	16 (55.2)
Ethnicity, *n* (%)	
Number (%) with data	29 (100)
Asian Indian	3 (10.3)
Asian Pakistani	1 (3.4)
Black Caribbean	2 (6.9)
White British	20 (69.0)
White Irish	1 (3.4)
White and Asian	1 (3.4)
Other	1 (3.4)
Index of multiple deprivation (score)	
Number (%) with data	6 (20.7)
Mean (SD)	11.6 (5.7)
Time since diabetes diagnosis (years)	
Number (%) with data	6 (20.7)
Mean (SD)	9.0 (4.9)
BMI (kg/m^2^)	
Number (%) with data	27 (93.1)
Mean (SD)	36.4 (7.7)
BMI (WHO international classification), *n* (%)	
Number (%) with data	27 (93.1)
Underweight (BMI <18.5 kg/m^2^)	0 (0.0)
Normal weight (BMI 18.5–24.9 kg/m^2^)	0 (0.0)
Overweight (BMI 25.0–29.9 kg/m^2^)	3 (11.1)
Obese (BMI ≥30.0 kg/m^2^)	24 (88.9)
Waist circumference (cm)	
Number (%) with data	21 (72.4)
Mean (SD)	119.0 (11.8)
Systolic blood pressure (mmHg)	
Number (%) with data	25 (86.2)
Mean (SD)	138 (23)
Diastolic blood pressure (mmHg)	
Number (%) with data	25 (86.2)
Mean (SD)	86 (12)

Participants' GPs were contacted to provide the following information:

- Data on SMI diagnosis
- Length of time since SMI diagnosis
- Length of time since T2D diagnosis
- Medical comorbidities
- Index of multiple deprivation score

Responses from the GP were received for six participants. In most cases, these were incomplete, making primary care record data the least complete data available.

### Data collection of diabetes and mental health outcomes and physical activity data

#### Data completeness

##### Diabetes outcomes

Twenty-two (75.9%) participants had valid HbA_1c_ data (Table 5). HbA1c values were considered invalid if they were obtained from routine blood test records that exceeded the 12-week window during which HbA1c reliably reflects mean blood glucose levels. Twenty-nine (100%) participants provided data for the Problem Areas in Diabetes Scale (PAID-5) and the foot care, smoking, and exercise questions on the Summary of Diabetes Self-Care Activities (SDSCA) self-report questionnaires. Several items of the SDSCA had missing data, including the general and specific diet questions (*n* = 28, 96.6%) and questions about blood glucose testing (*n* = 27, 93.1%).

**Table 5:** Data collection completion rates for diabetes and mental health outcomes.

Outcome, measure, or scale	Number (%) of participants with data available (total *n* = 29)
Diabetes outcomes	
HbA_1c_ (blood test)	22 (75.9)
Responded to the question about being prescribed insulin treatment	28 (96.6)
Diabetes distress: Problem Areas in Diabetes (PAID-5) Scale	29 (100)
Summary of Diabetes Self-Care Activities (SDSCA)	
General diet score	28 (96.6)
Specific diet score	28 (96.6)
Exercise score	29 (100)
Blood–glucose testing score	27 (93.1)
Foot care score	29 (100)
Smoked in the last 7 days	*29* (100)
Number of cigarettes smoked on an average day	7 (100)
Mental health outcomes	
Brief Psychiatric Rating Scale (BPRS)	29 (100)
Patient Health Questionnaire-9 (PHQ-9)	29 (100)

##### Mental health outcomes

All 29 participants provided complete data for mental health using the Brief Psychiatric Rating Scale (BPRS) and the Patient Health Questionnaire-9 (PHQ-9) (Table 5).

Further outcome data are summarised in Supplementary Appendix 7.

##### Physical activity data completion rates

A total of 29 participants (100%) completed the self-report International Physical Activity Questionnaire (IPAQ). At baseline assessment, eight participants were provided with a wrist-worn accelerometer to wear continuously (24 h per day) for 7 days. All participants returned the accelerometer after seven days. For one participant, no data could be downloaded; therefore, data were available for analysis for only seven participants (Table 6).

**Table 6:** Summary of accelerometer wear time for *n* = 7 participants.

	Weekdays	Weekend	All days
Number of valid days participants wore the accelerometer			
Mean (SD)	3.3 (1.0)	1.6 (0.8)	4.9 (1.6)
Valid wear time (hours per day)			
Mean (SD)	18.6 (3.0)	22.1 (4.6)	19.6 (3.4)

#### Summary of diabetes and mental health and physical activity outcomes

##### Diabetes outcomes

The mean HbA_1c_ for participants was 64 mmol/mol (SD 14.3); seven (24.1%) participants were treated with insulin. The mean PAID-5 score was 5.8 (SD 5.5), with 10 participants (34.5%) experiencing high levels of diabetes distress. Seven participants reported smoking in the previous 7 days, with a mean of 20.4 (SD 14.6) cigarettes smoked on an average day. Further details of the diabetes-specific outcome data can be found in Supplementary Table 1.

##### Mental health outcomes

The mean Brief Psychiatric Rating Scale score was 34.2 (SD 11.7), with almost half of the participants classified as moderately ill (*n* = 14, 48.3%). The mean PHQ-9 score was 12.8 (SD 6.5), with the largest proportion of participants reporting moderate depression (*n* = 9, 31.0%), followed by moderately severe depression (*n* = 8, 27.6%). Further details of the mental health-specific outcome data are shown in Supplementary Table 2.

##### Physical activity outcomes

According to the IPAQ questionnaire, 24 (82.8%) participants reported low activity, 4 (13.8%) participants reported moderate activity, and 1 (3.4%) reported high activity levels (Supplementary Table 3).

No participants (*n* = 0) used the Change One Thing mobile app, so no app usage data or Mechanisms of Action questionnaire data, which were to be collected through the mobile app, can be reported.

### Exploratory economic evaluation

#### Training and intervention delivery costs

Nineteen coaches were trained in three cohorts; six coaches withdrew. Training costs were £76,827 in total, or £3,841 per coach, or the equivalent of £2,649 per participant. For the 21 participants who received more than one session, the mean opportunity costs of staff in intervention delivery per participant were £1,827 (SD £1,336), ranging from £312 to £6,048 per participant. The workbook was priced at £8 per copy. The operation costs of the app were £1,817 over the 16-week intervention period.

#### Data collection completion rates for health/social care resource use and health-related quality of life

Self-report data (health and social care resource use questionnaire and EQ-5D-5L health-related quality of life questionnaire) were collected from 29 participants. Only six participants had information extracted from primary care medical records on health/social care resource use and medication for diabetes and SMI. The intention was that both self-report and primary care record data would be collected at baseline; however, data were extracted from the medical records 2–4 months later than the date of self-report.

Summary of participant-reported health-related quality of life and health/social care resource use outcomes

Except for one incomplete EQ-5D-5L, most (26) participants reported no problems with self-care and usual activities, while over half reported at least some problems with mobility, pain/discomfort, and anxiety/depression (Supplementary Table 4).

All but two participants accessed some type of primary care practice-based service, although video call consultations were rare. Seven participants reported using the accident and emergency (A&E) department at a hospital, with four being admitted. Four participants called the emergency number 999, and 11 used hospital-based services. Ten participants called the NHS non-emergency number 111, and none used NHS 111 online. Most of the listed community-based services were not used by most participants. No participants reported any use of NHS counselling or psychotherapists. Community mental health teams were the most frequently used service, accessed by 21 participants, with an average of 8.5 contacts (SD = 9.3), ranging from 1 to 30 contacts. Twelve participants had contact with a community psychiatric nurse, 10 with a pharmacist, 11 with an NHS optician, and 9 with an NHS dentist. There was considerable variation among those who used the same service. Individuals with high usage (value ≥ mean + 2xSD) appeared in several services. All 29 participants reported they were on medication for a ‘prolonged period’ (this was not defined specifically in the questionnaire and thus reflects the participant's interpretation of what ‘prolonged’ means). Except for three with missing information, the number of medicines that were currently taken by participants ranged from 0 to 26 (mean 9.1, SD 4.9). We observed some inconsistencies in self-reported medication use, as one participant who reported currently taking zero medicines also reported receiving medicine once a month in the last 2 months.

### Intervention participation

Six participants did not start the intervention; 23 participants received at least one session. One participant received a single session before withdrawing from the study. Analysing participants who received more than one session, the mean number of sessions participants received was 8.0 (SD 3.6). Of the 23 participants who started the intervention, 12 (52%) received eight sessions or more (at least 50% of intervention sessions). In terms of the progression criteria, as 66% of participants received at least one session, we fell short of the progression criteria (intervention sessions delivered to 80% of participants). Hence, modifications to study processes will be needed before moving forwards to the RCT.

The mean number of days during which participants received the intervention (from first session to withdrawal or last session) was 89.7 (SD 41.1), ranging from 23 to 170 days. The mean number of days between sessions was 15.1 (SD 20). The length of individual core sessions ranged from 3 min to 2 h.

#### Withdrawal from the intervention

Ten participants (34%) withdrew from the intervention following baseline data collection. Data on the reasons for withdrawal were available for eight participants. They included deterioration in mental health (*n* = 3); not feeling that they were benefiting from the intervention (*n* = 2); the intervention was too long (*n* = 1); not interested in participating in the intervention (*n* = 1); and disengagement (*n* = 1).

#### Session logs

Two hundred and thirteen sessions were recorded, including initial, core, and final sessions; 191 session logs were returned by coaches. 162 were core session logs where coaches documented participants' goals and the diabetes education topics covered. Among these, 113 logs (69.8%) contained valid responses documenting goals, and 91 logs (56.2%) included valid responses reporting the diabetes educational content. Most sessions involved setting goals related to healthy eating (*n* = 41, 36.2%), exercise (*n* = 27, 23.8%), and sleep (*n* = 22, 19.5%). In relation to the diabetes education content, most sessions where education was reported covered the topics ‘Why eat and drink healthier?’ (*n* = 21, 23.1%) and ‘Why be more physically active?’ (*n* = 10, 11%).

#### Adverse events

Six adverse events were reported during the study, including three participants contracting Covid-19: one further participant being hospitalised with a Covid-19 infection (serious adverse event) and two adverse events due to deteriorations in the participant's mental health.

### Intervention fidelity

Thirty-nine audio recordings were estimated to be available at the beginning of the feasibility phase [13 coaches × 3 recordings each (1 first session; 1 core session; and 1 final session) = 39 recordings]. Eleven recordings (3 first session, 5 core session, and 3 final session) from six coaches were received. Only 2 out of 13 coaches sent all three recordings. There were several reasons for this:

- There were challenges in setting up data transfer to the university from study sites due to variations in organisational policies and procedures.
- Some participants were unwilling to be recorded.
- Due to small participant numbers/withdrawals and location, some coaches were not able to deliver the requisite number of sessions to be recorded.

All 11 recordings were successfully measured using the IF tools, which were iteratively refined. This fulfilled our objective to demonstrate the feasibility of the IF measure for the definitive RCT, albeit the barriers to obtaining recordings will need to be addressed.

### Evaluation of training

Fifteen of the 19 coaches completed evaluation questionnaires. Eleven felt confident to deliver the weekly DIAMONDS sessions as per the coach manual, and ten felt confident to use the participant Change One Thing workbook, while only seven reported feeling confident to use the Change One Thing app. Two coaches reported that they had not received the training materials or coach manual prior to training. Positive feedback was received from coaches about the training, including comments that it had been ‘fantastically ran’, that coaches enjoyed the interactivity of the training through ‘sharing experiences', and felt they had ‘learnt new skills’. Suggested adaptations to training sessions included face-to-face delivery (due to the Covid-19 pandemic, training was delivered exclusively online), more time, and more roleplay to enable further opportunity to practice.

Eight coaches chose to have a mentorship call. These calls provided an opportunity for coaches to discuss what was going well, identify challenging areas, and explore solutions to any barriers encountered. Throughout the feasibility study, refinements to the training were made considering the feedback obtained.

## Discussion

### Summary of key findings

This feasibility study demonstrated that while recruitment targets for the DIAMONDS intervention for people with SMI were successfully met, challenges emerged in sustaining weekly session attendance. Although over half of the participants received at least eight sessions, strict adherence to weekly participation proved impractical, leading to adjustments for the planned RCT to allow greater flexibility in delivery. These attendance patterns illustrate the reality of working with people with SMI and physical comorbidities such as diabetes, whose health status can fluctuate substantially from week to week. A too rigidly scheduled approach is therefore unlikely to be sustainable or equitable, and it will be important to build in flexibility into how frequently the intervention is delivered so that it can fit around participants’ needs, preferences, and circumstances. High self-report data completion rates indicated the feasibility of participant-reported measures, though some physical and primary care data were less consistently collected, prompting refinements in data collection strategies with options to use routinely collected data where appropriate. Overall, the intervention was acceptable to both participants and coaches, and the development of a fidelity framework will support consistent delivery in the RCT.

### Study strengths and limitations

Due to the Covid-19 pandemic, research capacity in the NHS was reduced, necessitating a shift from the originally intended feasibility RCT to a single-group design. This adaptation allowed us to preserve essential opportunities to test the feasibility and acceptability of the DIAMONDS intervention, as well as the practicality of key study procedures, including recruitment and data collection, while minimising the burden on NHS resources. The internal pilot phase of the definitive RCT has been designed to test recruitment assumptions and trial procedures such as randomisation and treatment allocation under post-pandemic conditions, ensuring that the limitations of the single-group feasibility design are fully addressed. However, this design adjustment limited our ability to test trial-specific processes, such as randomisation and control group comparisons, which will be addressed in the subsequent RCT with a 12-month internal pilot phase, during which we will be able to establish robust procedures for recruitment, randomisation, and retention.

Despite these constraints, the study achieved robust recruitment and high data completion rates in self-reported measures, indicating strong engagement and feasibility in the SMI population. However, some challenges were encountered in collecting physical measures and primary care data, highlighting areas for refinement in the RCT. Recruitment was restricted to secondary care settings, and as such the findings may not fully represent the broader population of individuals with SMI, especially those managed solely in primary care or by third-sector services. The RCT will include recruitment from primary care and third-sector organisations to broaden access and evaluate these pathways in line with the original protocol.

The inclusion of an intervention fidelity framework is a key strength, as it provides a foundation for assessing and ensuring consistent delivery across settings in the RCT. Another strength lies in the exploratory economic evaluation conducted in this feasibility phase, which has provided valuable insights into resource allocation and cost-effectiveness considerations for the larger trial.

### Implications of feasibility study findings for RCT

In the context that the progression criteria for recruitment and the feasibility and acceptability of the intervention were met, the study steering committee reached a consensus decision that progression to the RCT was appropriate. Although the initial feasibility criteria for intervention participation were not fully achieved, this directly informed an important change in the DIAMONDS intervention for the RCT phase, specifically the decision to extend the intervention over six months without specifying the number of sessions. This adaptation allows for a more person-centred approach, enabling coaches to tailor the frequency and intensity of sessions according to participants' individual needs, rather than adhering to a rigidly structured schedule. This approach aligns with findings about the need to adopt flexible and person-centred approaches that accommodate individual variability to improve patient engagement and outcomes (27).

A striking finding was that none of the participants used the Change One Thing mobile app. This aligns with existing evidence that highlights challenges in adopting digital tools for diabetes self-management in the general population, including technical literacy, data privacy concerns, and difficulties with app usability (28). Despite these barriers, there is a significant emphasis in current UK health policy on transitioning from analogue to digital healthcare solutions (26). We explored in the intervention co-design whether a mobile app should be included as part of the intervention. While support for the app was not unanimous, approximately half of the service-user participants were in favour of including a digital component alongside the workbook. This highlights that digital approaches have the potential to appeal to people with SMI and should not be ignored. To respond to these policy imperatives and to support equity and digital inclusion among people with SMI, we remain committed to exploring the use of the Change One Thing mobile app in the main trial. In discussion with the Programme Management Team and the Programme Steering Committee, a decision was made to continue using the app in the RCT, with an emphasis on an improved onboarding process and a greater emphasis on the training around the app for the coaches. Due to unforeseen delays in the completion of the app prior to the start of the feasibility study, the coach training focused primarily on the workbook, rather than the app. This is likely to have affected coaches’ confidence in using the app with participants. The lack of engagement with the app also reflects wider issues of digital exclusion and variability in technological confidence among people with SMI (29). By including supported onboarding and coach-facilitated demonstrations, the RCT will be able to determine whether structured digital support can improve equitable participation rather than assuming uniform digital readiness.

Owing to restrictions during the Covid-19 pandemic, the group support sessions were not held as planned. Given that the intervention was successfully delivered using one-to-one sessions only, group sessions were not included in the intervention design for the RCT. This decision was made in agreement with the Programme Management Team and DIAMONDS Voice. Group diabetes self-management sessions have been associated with the smallest reductions in HbA1c in the general population (30), and offering personalised and tailored self-management support is crucial for effectively managing complex long-term conditions (31).

Several refinements to coach training were made in response to feedback during the feasibility study, which have been taken forward to the RCT. These include improving the coach recruitment process, including sending materials and pre-training work earlier, change to timing of training delivery to enable completion of self-directed learning, face-to-face training, and provision of booster training. We will develop a standard operating procedure for the use of recordings to remind coaches of key protocols. During site setup, we will also discuss IT and data transfer requirements to identify and address barriers early on.

In the feasibility study, data collection from primary care records proved to be time-consuming and logistically difficult, resulting in a low completion rate. Consequently, in the RCT, reduced data items are being collected from medical records, and these will be collected by the R&D teams in secondary care. HbA_1c_ levels, which represent average blood glucose over the past 2–3 months, are relatively stable. Using the most recent measurement, even if not taken specifically for the study, can provide a valid estimate. For example, in population health studies and clinical audits, routinely collected HbA_1c_ data are commonly used and have been shown to correlate well with primary outcome measures (32). To maximise completion rates of the primary outcome (HbA_1c_) in the RCT, participants who decline a blood test or where blood taking is not possible for other reasons will therefore be asked to consent to sharing the results of their most recent routine blood test results in primary or secondary care records. Collecting this information through routine data options is expected to improve data quality and reduce missing data, while maintaining feasibility for busy clinical teams. As participant self-report achieved high completion rates, this method has been selected for the collection of care service resource use data in the RCT.

Clinical information about the sample included in this feasibility study, such as SMI diagnosis or duration of diabetes, was limited as we only attempted to collect this information from primary care records. Due to the difficulties we encountered with this, we have changed our eligibility screening forms for the RCT, where this information will be collected in secondary care (if needed, with input from primary care notes) at the point of a participant’s first entering the study.

Taken together, the feasibility findings highlight the complexity but potential of implementing structured diabetes self-management interventions for people with SMI. This was a real-world and pragmatic evaluation, and as such, the challenges encountered, such as variable attendance, incomplete physiological data, and limited digital engagement, represent authentic delivery conditions rather than methodological flaws. Each has directly informed improvements for the RCT design, which offers adaptable scheduling, options to use routine health records, and enhanced digital and coaching support.

## Conclusions

This feasibility study underscores both the promise and complexity of delivering a structured diabetes self-management intervention to individuals with SMI. While recruitment success reflects a strong interest and need for targeted diabetes support in this population, the challenges encountered in achieving regular session attendance highlight the importance of flexibility in intervention design. Our findings point towards the need for adaptable delivery models that can accommodate the challenging and often variable needs of individuals with SMI. Additionally, the discrepancies in data completion across self-reported, physical, and primary care measures highlight the need for a streamlined, patient-centred approach to data collection that minimises burden on recruiting sites and research staff. The refined fidelity framework and a more flexible intervention delivery approach are expected to support both adherence and consistency in the RCT, ultimately contributing to a more robust understanding of how to effectively support diabetes self-management for people with SMI.

## Data Availability

The raw data supporting the conclusions of this article will be made available by the authors, without undue reservation.
