# Supplementary material for: The DIAMONDS intervention for type 2 diabetes for people with severe mental illness: findings from a single-group feasibility study
Source: Front Health Serv. 2025 Nov 26;5:1688787. doi: 10.3389/frhs.2025.1688787 (PMC12689556; doi:10.3389/frhs.2025.1688787)
Supplement: Supplementary file 1 [file Datasheet1.pdf]

UNIVERSITY of York | NHS Bradford District Care NHS Foundation Trust | NIHR National Institute for Health Research

diamonds.

We invite you to take part in a research study

We are looking at a new support programme for people who have type 2 diabetes and a mental illness. The programme aims to help people to manage their conditions. It has been created in partnership with people who have these conditions.

We are inviting adults to take part who have been diagnosed with:	**Type 2 diabetes**	*and*	**a mental illness such as:**<br>**Bipolar disorder**<br>**Schizophrenia**<br>**Schizoaffective disorder**

Key things you need to know

- We are running the support programme to find out what people think about it and to test our research methods.
- Before you decide whether to take part, we want to tell you more about why the research is being done and what it will involve. **Please read through this information sheet for more details about the study.** You can discuss the study with others if you wish, such as friends, relatives, your GP, diabetes nurse, or mental health service provider.
- It is entirely up to you whether or not you take part. If you choose not to take part this will not affect the care or any benefits you currently receive.
- **A member of the research team will contact you in the next few days to discuss the study.** Please feel free to contact us in the meantime with any questions you may have, or if you would like any further information.

How to get in touch with us

If you have any questions about the study, you can contact Jennifer Brown, Programme Manager:

01904 321662 or 07384 239004 or jennifer.brown@york.ac.uk

A friend or relative may speak to us on your behalf if you wish. There is an answering machine available 24 hours a day, so please leave a message and we will return your call as soon as possible.

We also have a website that includes information about the study: www.diamondscollaboration.org.uk

Contents

1 Why we are doing the study?

2 Why am I being asked to take part?

3 How does the study work?

4 What will happen to me if I take part?

5 Possible benefits and disadvantages of taking part

6 More information about taking part

7 What do I need to do now?

1 Why are we doing the study?

What are we studying?

The study is part of a research programme research that aims to help people manage diabetes and a mental illness. Managing diabetes involves doing things like taking medications, monitoring symptoms, preventing complications, and leading a healthy lifestyle. This is called 'self-management' because it is about doing things to look after yourself.

Why do we think this is important?

People living with a mental illness are more likely to have diabetes than other people. Diabetes self-management can be harder for people with a mental illness. This can be for different reasons.

Diabetes support programmes are available in the NHS but they may not be suitable for people who also have a mental illness. We have created a new support programme called DIAMONDS to address some of the difficulties involved in self-management of these conditions. DIAMONDS was created in partnership with people who have diabetes and a mental illness.

What do we need to do?

The study is to find out whether we can deliver the new support programme and whether it is acceptable to the people running it and receiving it. We will also test our research methods so that we can design a larger study in the future to find out whether the new support programme improves people's health.

2 Why am I being asked to take part?

You have been identified as someone who lives with diabetes and a mental illness (such as bipolar disorder, schizophrenia, or schizoaffective disorder). You may have been given an information pack by your general practice or community mental health team and sent us a form with your contact details. You may have seen posters advertising the study and contacted the study team directly. We have also contacted some people who have joined a research register and agreed to be contacted about relevant research studies. The research team have used your name and contact details to provide you with this information about the research study.

3 How does the study work?

How do we find out whether we can deliver the new support programme?

We need to practise running the new support programme. This is called a "feasibility study". We need to test our research methods to prepare for a much larger study in the future. We hope that 30 people will take part in this study.

4 What will happen to me if I take part?

We will contact you again in a few days' time to discuss the study with you. You can also contact the study team directly to let them know you are interested in taking part. Our contact details are on page 1.

When you speak with a researcher you can ask them any questions you have about the study. You do not have to take part in the study.

The researcher will check you are eligible to take part in the study. If you are not eligible, or if the study isn't quite right for you, your involvement will finish at this point. The researcher will explain this to you.

If you are eligible to take part and you are happy to proceed with the study, we will ask your local NHS Trust to contact you. They will arrange an appointment with a researcher.

Researcher appointment

The meeting will be at a local clinic. You can choose when it takes place and you can have someone with you if you like. Some parts can also be done over the phone. The researcher will discuss this with you and you will decide together what will work best. The appointment will last no longer than 60 to 90 minutes.

The researcher will:

- Explain the study.
- Answer any questions you may have.
- Check you understand what the study involves.
- Ask you to sign a consent form.
- Ask you to fill in a questionnaire about your health.
- Take a blood sample from you.
- Take some measurements such as your height, weight, and blood pressure.

Some people will be asked to wear a special kind of wrist-band (a bit like a digital watch). It measures if you are moving or staying still throughout the day. It does not track where you are or record any other information. The researcher will explain this to you and you do not have to agree to wear it. If you do agree, you will be asked to wear the wrist-band for one week and to then return it to us.

You will be asked to wear a small device that measures your blood sugar (also called "glucose"). The researcher will explain this to you and you do not have to agree to wear it. If you do agree, you will be asked to wear the device for two weeks and to then return it to us. You don't have to agree to wear any of these devices and you can stop wearing them at any time if you change your mind.

DIAMONDS

The DIAMONDS programme aims to help with self-management of diabetes and a mental illness. This will be in addition to the care you normally receive.

The DIAMONDS support programme involves weekly appointments, monthly support sessions, and daily use of a workbook or digital app.

It will involve you working with someone called a DIAMONDS Coach. The Coach will be trained in delivering the new support programme and will have experience of supporting people living with a mental illness.

A member of the team will contact you to arrange a convenient time for your DIAMONDS Coach to meet you to support your diabetes self-management. This might happen in person or on the phone.

Weekly appointments

The first appointment will last between 60-90 minutes. You can choose where this meeting takes place. The DIAMONDS Coach will work with you to decide which parts of diabetes self-management to support you with. They will make a plan to help you try to change one thing about your self-management, for example sleeping or taking medication. You will then try to carry out that plan during the first week.

You will speak to your DIAMONDS Coach once a week for the rest of the 16-week support programme. In these appointments the Coach will provide information about diabetes and support you to make healthy lifestyle changes. They will review how you get on with your self-management plans and find ways to overcome any problems you may have had with following the plans.

These appointments can vary in length but may last between 30 and 60 minutes. These meetings can be by telephone or video call.

Daily use of paper-based workbook or digital app

In between the weekly appointments you will use a workbook or a digital app every day to help you with your self-management plans. The DIAMONDS Coach will set you up with the workbook or app and they will support you with using it. The workbook and app can:

- Remind you to do important things such as take your medication.
- Give you information about the benefits of self-management.
- Suggest activities you can do to improve your mood.
- Log how you are getting on with your chosen self-management plans.

In the weekly appointments, the DIAMONDS Coach will discuss the workbook or digital app with you to see how you are getting on. They will record some of this information to help us better understand how the app and workbook are being used.

You will have the option to use the digital app on your own smart phone alongside the workbook. Your DIAMONDS Coach will help you download and install the app and set it up with you. You don't have to decide right away if you want to use the app; you can start using it at any time during the 16 weeks. You can also stop using the app at any point. This will not affect your participation in the study or the care you are receiving.

You do not have to use the digital app if you do not want to. The paper-based workbook can be used instead to do the same tasks.

Monthly group support sessions

As well as the weekly meetings and workbook/app, there will be monthly group support sessions that you can attend if you want to. There will be about 10 other DIAMONDS participants in the sessions. The sessions will be held in your local area in the afternoon and will last 90 minutes. Lunch will be provided. There will be four group sessions during the support programme. The sessions will be run by DIAMONDS Coaches.

During the group sessions the Coaches will give people the chance to talk about any problems or successes they have had with the support programme. You do not have to talk about your own problems if you do not want to.

Not everybody wants to attend group sessions but many people find it helpful to meet other people who are on the same support programme. Being in a diabetes support group with other people who have a mental illness can be easier than being in a general diabetes support group.

As we will be running this support programme for the first time, there may be a researcher present at some of the group sessions to check on how it is going. With your consent, there may be a video camera recording some of the weekly meetings and monthly group sessions. This is so that we can check on what support the DIAMONDS Coaches offer and how people engage with it.

At the end of the study we will ask a small number of people to speak with a researcher about their experiences of receiving the support programme. If we ask you to do this, we will give you more information about what it would involve. If you agree to do this we will ask you to fill in a separate consent form. If you do not wish to do this, you can still take part in the rest of the study.

What are the possible benefits of taking part?

Taking part could help improve future support for people who have diabetes and a mental illness. Although we cannot promise that taking part in the study will help you, you may learn more about your diabetes which could help you to manage this better.

What are the possible disadvantages of taking part?

Taking part in the study will require you to attend appointments, have body measurements and blood taken, and complete a study questionnaire which will take time. Taking part in this study will not require you to travel further than a local NHS site.

During the ongoing Covid-19 (coronavirus) pandemic we might have to make changes to the support programme to ensure that everyone is kept safe. For example, it might not be possible to meet your DIAMONDS Coach in person or to attend a group session. Your Coach and any researchers you have contact with will discuss this with you. You can get in touch with the research team at any time if you have questions or concerns. Our contact details are on page 1.

6 More information about taking part

Do I have to take part?

No, it is entirely up to you to decide whether to take part or not. Please feel free to contact us if you would like more information, or to ask us any questions. Our contact details are on page 1. Please also discuss the study with family or friends if you wish.

You will be asked to sign a consent form if you decide to take part to confirm that you are happy to participate in the study and that you understand what is involved. We will give you a copy of this for you to keep. Please also keep this information sheet. Even if you agree to take part in the study, you are free to stop at any time, without giving a reason. We will still use the information we have received from you up to the point you stop taking part.

The care or any benefits you normally receive will not change if you decide not to take part, or if you decide to stop taking part once you have joined the study.

Will I receive any payment for taking part?

We will pay all postage and travel costs in relation to the study. We would also like to offer you a £10 gift voucher for completing the study questionnaire. You do not have to accept this.

Will my GP be involved?

We will tell your GP if you agree to take part in the study. We will also ask for your permission to get in touch with your GP if we have any concerns about your health whilst you are taking part in the study. You can contact your GP for support at any time during your involvement in the study.

What will happen to information collected about me during the study?

We will treat any information you give us in confidence. We will store all of your information safely and securely. We will not mention your name in any publications about the study. We will make sure that no individuals can be identified in the study results.

Sometimes we are asked to share information we collect in studies with other researchers who are looking at similar ways to improve healthcare. With your agreement, we would like to be able to share the information we collect about you as part of this study. We will always remove all identifiable information before we share your information. This is controlled by strict University of York and NHS Trust procedures.

Research which is similar to this study might be carried out in the future, either by us or by other researchers. With your agreement, we would like to send you information about these related studies. The information will only be sent to you by a member of our study team. We will not pass your personal details to anyone outside of the

study team. This would be controlled by strict University of York and NHS Trust procedures. You can choose whether or not you agree to this when you fill in your consent form.

We will safely keep the information you give us as part of the current study for 10 years after the study ends. It will then be destroyed securely. Paper records will be handled by the study team and kept in locked cabinets. Electronic records will be held on a secure computer server approved by the University of York. Notes on DIAMONDS support sessions will be held in accordance with the service delivering the DIAMONDS support and in line with University of York and NHS Trust procedures. These will always be confidential and secure. Only authorised members of the study team will have access to your information.

The study team would be happy to answer any questions you may have about how we use the information we receive from you as part of the study.

What if there is a problem?

If you have concerns about the study or if there is anything you would like to ask about the study, please get in touch with us. Our contact details are on page 1. If you would prefer you may contact the Chief Investigator: Prof Najma Siddiqi, Tel 01904 321681, email: najma.siddiqi@york.ac.uk.

It is not anticipated that any harm will to come to you from being in this study. However, if you wish to complain or have any concerns about any aspect of the way you have been approached or treated during the course of the study, the normal NHS complaints processes are available to you through your local Patient Advice and Liaison Service Tel:

Tel: 01274 251440

Email: advice.complaints@bdct.nhs.uk

If you wish to raise a complaint on how we have handled your personal data, you can contact the University of York's Data Protection Officer who will investigate the matter (dataprotection@york.ac.uk). If you are not satisfied with our response or believe we are processing your personal data in a way that is not lawful you can complain to the Information Commissioner's Office (ICO) whose details can be found here: https://ico.org.uk/.

What will happen to the results of the study?

When the study has finished and the results have been published, we will send you a summary of the study results if you agree for us to do so. You can contact your GP practice or the study team directly if you decide not to take part in the study, but would still like to receive a summary of the results. We will also keep our study website up to date with news about our research.

Who is organising and funding the study?

This study is organised by Bradford District Care NHS Foundation Trust and the Universities of York, Southampton, Leicester and Leeds. This group is working with NHS Trusts and GP practices in England.

The study has been funded by the Department of Health's National Institute for Health Research Programme Grants for Applied Research programme.

The study has been reviewed by an independent group of people, called a Research Ethics Committee. This is to protect your safety, rights, wellbeing and dignity. This study was looked at and approved by the Leeds West Research Ethics Committee, reference 21/YH/0059.

A patient involvement group called DIAMONDS Voice has contributed to development of the study and will continue to support the study.

Are there any independent organisations I can contact?

For general information about research you might wish to contact INVOLVE (Tel. 023 8059 5628). For information about diabetes you may wish to contact Diabetes UK (diabetes.org.uk or Tel. 0345 123 2399). For more general information about mental illness, you may wish to contact Rethink (rethink.org or Tel. 0300 5000 927).

---

7 What do I need to do now?

What do I need to do if I would like to take part in the study?

You can either wait until we contact you in the next few days or you can contact us (see page 1).

Do I need to do anything if I don't want to take part in the study?

Simply tell us that you do not want to take part when we contact you within the next few days. You will not have to give a reason for this. The care you receive will not be affected in any way.

Please turn over.

Data Protection statement

The University of York is the sponsor for this study based in the United Kingdom. We will be using information from you in order to undertake this study and will act as the data controller for this study. This means that we are responsible for looking after your information and using it properly.

The University of York is a publicly funded organisation that conducts research to improve health, care and services. Research following the UK Policy Framework for Health and Social Care Research is conducted to serve the interests of society as a whole. This means that the University of York is using the legal basis provided under the Data Protection Act 2018 and the General Data Protection Regulation of 'for the purposes of research conducted in the public interest' to use your personal data for this research.

Your rights to access, change or move your information are limited, as we need to manage your information in specific ways in order for the research to be reliable and accurate. If you withdraw from the study, we will keep the information about you that we have already obtained. To safeguard your rights, we will use the minimum personally-identifiable information possible.

You can find out more about how we use your information at:

https://www.york.ac.uk/healthsciences/research/trials/trials-gdpr/

https://www.york.ac.uk/healthsciences/research/trials/trials-gdpr/research-partcipants/

Participating NHS bodies and The University of York will collect information from you for this research study in accordance with the sponsor's instructions.
The University of York will use your name and contact details to contact you about the research study, and make sure that relevant information about the study is recorded for your care, and to oversee the quality of the study. The University of York will pass these details to the participating trust along with the information collected from you. The only people in the participating trust who will have access to information that identifies you will be people who need to contact you about the research you are taking part in, to invite you to take part in other research studies you may be interested in or audit the data collection process. The people who analyse the information will not be able to identify you and will not be able to find out your name or contact details. Individuals from University of York and regulatory organisations may look at your medical and research records to check the accuracy of the research study.
The University of York will keep consent forms and other information about you from this study for 10 years after the study has finish. Contact details will be kept for three years unless you have given consent to be contacted about future research.

Thank you for reading this information.
