# Supplementary material for: The DIAMONDS intervention for type 2 diabetes for people with severe mental illness: findings from a single-group feasibility study
Source: Front Health Serv. 2025 Nov 26;5:1688787. doi: 10.3389/frhs.2025.1688787 (PMC12689556; doi:10.3389/frhs.2025.1688787)
Supplement: Supplementary file 2 [file Datasheet2.pdf]

UNIVERSITY of York

NHS Bradford District Care NHS Foundation Trust

NIHR | National Institute for Health Research

diamonds.

PARTICIPANT CONSENT FORM
Title of Study: DIAMONDS
IRAS ID: 279019
REC Reference: Leeds West REC 21/YH/0059
Participant ID:

If you wish to take part in the DIAMONDS study, **please place your initials in each of the boxes below, sign and date this form.**

*Please **initial** each box*

1. I confirm that I have read and understand the information sheet version v1.7 dated 14/06/2021 for the above study and have had the opportunity to ask any questions about the study and any questions have been answered to my satisfaction. Initials
2. I understand that my participation is voluntary and that I am free to withdraw at any time without giving any reason, and without my medical care or legal rights being affected. Initials
3. I agree to the University of York holding copies of my consent form, other study related documents and my contact details to allow them to contact me for appointments and send me letters and questionnaires. Initials
4. I agree to my GP being informed of my participation in the study and being advised of any significant information relating to my health that comes to light during my participation. Initials
5. I understand that relevant sections of my hospital/GP medical notes and data collected during the study, may be looked at by individuals from the University of York, from regulatory authorities or from the NHS Trust, where it is relevant to my taking part in this research. I give permission for these individuals to have access to my records. Initials
6. I understand that the information collected about me could be used to support other research in the future, and may be shared anonymously with other researchers. Initials
7. I understand that the information held and maintained by Bradford District Care and Foundation Trust and other central UK NHS bodies may be used to help contact me or provide information about my health status. Initials
8. **I agree to take part in the DIAMONDS Study.** Initials

In addition to the above statements please initial the following boxes to indicate whether you agree with the following statements. Your participation in this research study will not be affected if you do not agree with these statements.

	Yes	No
I am happy to be contacted about giving feedback on the DIAMONDS study	Initials	Initials
I would like to receive a summary of the study findings	Initials	Initials

Print name	d d / m m / y y y y<br>/ / 2 0	Signature
Name of participant (*please print*)	Date	Signature of participant
Print name	d d / m m / y y y y<br>/ / 2 0	Signature
Name of person taking consent (*please print*)	Date	Signature of person taking consent

[Original to be kept in Trial Master File; one copy given to participant; one copy sent to participant's GP]
