# Supplementary material for: The DIAMONDS intervention for type 2 diabetes for people with severe mental illness: findings from a single-group feasibility study
Source: Front Health Serv. 2025 Nov 26;5:1688787. doi: 10.3389/frhs.2025.1688787 (PMC12689556; doi:10.3389/frhs.2025.1688787)
Supplement: Supplementary file 3 [file Table1.docx]

# Appendix 1 – CONSORT checklist


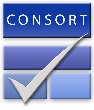
CONSORT 2010 checklist of information to include when reporting a pilot or feasibility trial*

| Section/Topic | Item No | | Checklist item | Adaptations for non-randomised feasibility study | Reported on page No |
| --- | --- | --- | --- | --- | --- |
|  | | Title and abstract | | | |
|  | 1a | | Identification as a pilot or feasibility randomised trial in the title | Identification as single group feasibility study | 1 |
|  | 1b | | Structured summary of pilot trial design, methods, results, and conclusions (for specific guidance see CONSORT abstract extension for pilot trials) | N/A | 2 |
|  | | Introduction | | | |
| Background and objectives | 2a | | Scientific background and explanation of rationale for future definitive trial, and reasons for randomised pilot trial | Reasons for feasibility study | 3 |
|  | 2b | | Specific objectives or research questions for pilot trial | Specific objectives for feasibility study | 4 |
|  | | Methods | | | |
| Trial design | 3a | | Description of pilot trial design (such as parallel, factorial) including allocation ratio | Description of single group feasibility study design | 4 |
|  | 3b | | Important changes to methods after pilot trial commencement (such as eligibility criteria), with reasons | N/A | Appendix 3 |
| Participants | 4a | | Eligibility criteria for participants | N/A | 5/6 (table 2) |
|  | 4b | | Settings and locations where the data were collected | N/A | 5 |
|  | 4c | | How participants were identified and consented | N/A | 7 |
| Interventions | 5 | | The interventions for each group with sufficient details to allow replication, including how and when they were actually administered | Intervention for single group | 7/8 |
| Outcomes | 6a | | Completely defined prespecified assessments or measurements to address each pilot trial objective specified in 2b, including how and when they were assessed | N/A | 9/10 (table 3) |
|  | 6b | | Any changes to pilot trial assessments or measurements after the pilot trial commenced, with reasons | N/A | 10 |
|  | 6c | | If applicable, prespecified criteria used to judge whether, or how, to proceed with future definitive trial | N/A | 15 |
| Sample size | 7a | | Rationale for numbers in the pilot trial | N/A | 6 |
|  | 7b | | When applicable, explanation of any interim analyses and stopping guidelines | N/A | N/A |
| Randomisation: |  | |  |  |  |
| Sequence  generation | 8a | | Method used to generate the random allocation sequence | N/A | N/A |
|  | 8b | | Type of randomisation(s); details of any restriction (such as blocking and block size) | N/A | N/A |
| Allocation  concealment  mechanism | 9 | | Mechanism used to implement the random allocation sequence (such as sequentially numbered containers), describing any steps taken to conceal the sequence until interventions were assigned | N/A | N/A |
| Implementation | 10 | | Who generated the random allocation sequence, who enrolled participants, and who assigned participants to interventions | N/A | N/A |
| Blinding | 11a | | If done, who was blinded after assignment to interventions (for example, participants, care providers, those assessing outcomes) and how | N/A | N/A |
|  | 11b | | If relevant, description of the similarity of interventions | N/A | N/A |
| Statistical methods | 12 | | Methods used to address each pilot trial objective whether qualitative or quantitative | N/A | 8-14 |
|  | | Results | | | |
| Participant flow (a diagram is strongly recommended) | 13a | | For each group, the numbers of participants who were approached and/or assessed for eligibility, randomly assigned, received intended treatment, and were assessed for each objective | Reported for whole sample | 15 |
|  | 13b | | For each group, losses and exclusions after randomisation, together with reasons | Reported for whole sample | 15 |
| Recruitment | 14a | | Dates defining the periods of recruitment and follow-up | N/A | 15 |
|  | 14b | | Why the pilot trial ended or was stopped | N/A | N/A |
| Baseline data | 15 | | A table showing baseline demographic and clinical characteristics for each group | N/A | 17/18 (table 4) |
| Numbers analysed | 16 | | For each objective, number of participants (denominator) included in each analysis. If relevant, these numbers should be by randomised group | N/A | 17/18 (table 4) |
| Outcomes and estimation | 17 | | For each objective, results including expressions of uncertainty (such as 95% confidence interval) for any estimates. If relevant, these results should be by randomised group | N/A | N/A |
| Ancillary analyses | 18 | | Results of any other analyses performed that could be used to inform the future definitive trial | N/A | N/A |
| Harms | 19 | | All important harms or unintended effects in each group (for specific guidance see CONSORT for harms) | N/A | 24 |
|  | 19a | | If relevant, other important unintended consequences | N/A | N/A |
|  | | Discussion | | | |
| Limitations | 20 | | Pilot trial limitations, addressing sources of potential bias and remaining uncertainty about feasibility | N/A | 26/27 |
| Generalisability | 21 | | Generalisability (applicability) of pilot trial methods and findings to future definitive trial and other studies | N/A | 27-29 |
| Interpretation | 22 | | Interpretation consistent with pilot trial objectives and findings, balancing potential benefits and harms, and considering other relevant evidence | N/A | 29/30 |
|  | 22a | | Implications for progression from pilot to future definitive trial, including any proposed amendments | N/A | 27-29 |
| Other information | | | |  |  |
| Registration | 23 | | Registration number for pilot trial and name of trial registry | N/A | 4 |
| Protocol | 24 | | Where the pilot trial protocol can be accessed, if available | N/A | 4 |
| Funding | 25 | | Sources of funding and other support (such as supply of drugs), role of funders | N/A | 30 |
|  | 26 | | Ethical approval or approval by research review committee, confirmed with reference number | N/A | 4 |

Citation: Eldridge SM, Chan CL, Campbell MJ, Bond CM, Hopewell S, Thabane L, et al. CONSORT 2010 statement: extension to randomised pilot and feasibility trials. BMJ. 2016;355. This is an Open Access article distributed in accordance with the terms of the Creative Commons Attribution (CC BY 3.0) license (<http://creativecommons.org/licenses/by/3.0/>), which permits others to distribute, remix, adapt and build upon this work, for commercial use, provided the original work is properly cited.

*We strongly recommend reading this statement in conjunction with the CONSORT 2010, extension to randomised pilot and feasibility trials, Explanation and Elaboration for important clarifications on all the items. If relevant, we also recommend reading CONSORT extensions for cluster randomised trials, non-inferiority and equivalence trials, non-pharmacological treatments, herbal interventions, and pragmatic trials. Additional extensions are forthcoming: for those and for up-to-date references relevant to this checklist, see [www.consort-statement.org](http://www.consort-statement.org).

# Appendix 2 – Study oversight and governance structures

Study sponsorship

Bradford District Care NHS Foundation Trust acted as the lead organisation and contractual partner with the NIHR and as such held overall responsibility for the delivery of the programme. The University of York acted as the sponsor for this feasibility study.

The DIAMONDS Programme Management Team

This feasibility study formed part of the DIAMONDS research programme, which was led by a Professor of Psychiatry as the Chief Investigator. The day-to-day running of the programme, including the feasibility study, was overseen by the Programme Manager with support from the workstream leads, who were experts in health services research methods and diabetes, respectively. Statistical oversight was provided by a Professor of Trials and Statistics.

Decisions about the feasibility study and the programme as a whole were made by the Programme Management Team, which included experts in diabetes, mental health, statistics, and health economics, as well as GPs, psychologists, and service user and carer representatives.

The DIAMONDS Programme Steering Committee

The Programme Management Team was accountable to and supported by the Programme Steering Committee, which also fulfilled the role of the Data and Ethics Committee (DMEC) for the feasibility study. The Steering Committee membership consisted of an academic GP, a trialist, a statistician, a psychiatrist, a diabetologist, and a service user representative.

Monitoring adverse events

*Definitions*

An adverse event was any unexpected effect or untoward clinical event affecting the participant. It could have been directly related, possibly related, or completely unrelated to the intervention. It could also have been classified according to severity, such that a non-serious Adverse Event (AE) included discomfort or slight worsening of symptoms, or a Serious Adverse Event (SAE), which might have been particularly harmful, dangerous, or required hospitalisation.

Hospitalisations for treatment planned prior to enrolment and hospitalisation for elective treatment of a pre-existing condition were not considered as an SAE. Complications occurring during such hospitalisation were AEs.

*Detecting and recording AEs and SAEs*

Any AEs or SAEs were reported to the Chief Investigator and were reviewed by a clinician independent of the DIAMONDS study team. This included all reported cases of COVID-19. The reporting period was from study entry up to the last follow-up visit. Details about AEs/SAEs were captured at each contact with a DIAMONDS Coach or during study assessments. AEs/SAEs that might have occurred since the previous visit or assessment were elicited from the patient by open questioning and recorded. All events related to the DIAMONDS intervention were recorded on adverse events forms. Further information could be requested for follow-up of these events. Detailed records were kept of all adverse events.

*Evaluation of AEs and SAEs*

Adverse events that were deemed possibly, probably or definitely related to participation in this study and all SAEs were evaluated for seriousness, causality, severity and expectedness by the chief investigator and reviewed by an independent clinician/mental health specialist. All AEs/SAEs were reviewed in terms of suspected causal relationship (e.g. unrelated, unlikely, possibly, probably, definitely) to the study intervention.

*Reporting AEs and SAEs*

All SAEs were reported to the sponsor and to the Research Ethics Committee (REC) in line with their guidelines. Serious events that were deemed unexpected and related events were usually reported to the REC within 15 days of the event being reported. All others were reported in the usual 6-monthly progress report. Any relevant further information was subsequently communicated, and events were followed up until the event was resolved or a decision was made that no further follow-up was necessary. In addition, all associated investigators were notified. The numbers and details of all AEs and SAEs were reported to the PMT and PSC.

AEs reported by study participants that were not classified as an SAE were reported and included in reports submitted to the PSC in agreement with the committee chair.

Where repeated adverse events (serious or non-serious) of a similar type were observed, these were discussed with the PMT and other relevant groups and were onward reported to the REC and Sponsor should concerns be raised in relation to the type of event and/or frequency observed.

Suicide and self-harm risk management

Inherent in the population under scrutiny was the risk of self-harm and suicide. We followed good clinical practice and adhered to a Risk Protocol for the monitoring of suicide and self-harm risk during all encounters with study participants. The study team had a wealth of experience in developing and implementing risk protocols for use in studies involving psychological interventions for SMI. When any risk to participants, due to expressed thoughts of self-harm or suicide, was encountered, a risk assessment was conducted. The level of risk was determined and discussed with a clinical member of the study team. Risk was reported to the participant’s GP (with the participant’s consent) where deemed necessary by a clinical member of the research team. Acute risk was dealt with immediately and involved a clinical member of the DIAMONDS study team. At least one clinical member of the team was on call at all times to respond to risk while participants were involved in the study. All members of the study team, DIAMONDS Coaches, and members of R&D teams involved in data collection at participating trusts completed training on the risk protocol, delivered by a clinical member of the team, before commencing contact with participants. Members of the study team, DIAMONDS Coaches, and R&D staff were provided with support and debriefing following risk if required.

Duty of care

We used YTU standard operating procedures to support researchers, DIAMONDS Coaches, and R&D staff in reporting to GPs or responsible services instances where there were concerns about the health of the participant.

A distress protocol had been developed and was followed in case any participants displayed signs of distress during the qualitative interviews. Participant wellbeing was the main priority and researchers conducting the interviews sign-posted further support as needed.

Researcher safety and lone working

Researcher safety was of paramount importance. We used the York Trials Unit (YTU) standard operating procedures for fieldwork and lone working (see Appendix 1). Fieldwork was defined as any research activity that involved data collection either on-site (university premises) or off-site (e.g. patient’s homes, hospital premises, and community centers). All researchers tasked with fieldwork undertook lone worker training and conducted a risk assessment with their line manager about the specific tasks to be carried out. Researchers were able to appoint a designated person (academic or administrative staff) who acted as a safety contact. A system was agreed between the researcher and the designated person to communicate when the fieldwork trip had started and finished. Researchers had regular debriefs with their line manager and the Chief Investigator to review this process and check that it was fit for purpose. Details of the lone worker policy were included in the researcher handbook.

Statement of indemnity and complaint handling

Normal NHS indemnity procedures applied. The University of York also provided relevant cover. The PIS provided participants with contact details of the Sponsor in case of complaint. If there was negligent harm during the study, when the NHS Trust owed a duty of care to the person harmed, NHS indemnity covered NHS staff and medical academic staff with honorary contracts only when the feasibility study had been approved by the R&D department. NHS indemnity did not offer no-fault compensation and was unable to agree in advance to pay compensation for non-negligent harm.

Monitoring quality control and assurance

Quality control was maintained through adherence to Standard Operating Procedures (SOPs), study protocol, the principles of ICH/GCP, research governance and relevant study regulations. The intervention delivered in this feasibility study was low risk and major safety data were not anticipated. Monitoring of study conduct and data collected was performed by a combination of central review and site monitoring visits to ensure the study was conducted in accordance with good clinical practice. The main areas of focus included consent, serious adverse events, and essential study documents. All monitoring findings were reported and followed up with the appropriate persons in a timely manner. The study was subject to inspection and audit by the University of York under their remit as sponsor and other regulatory bodies to ensure adherence to GCP. The investigator(s)/institutions permitted study-related monitoring, audits, REC review and regulatory inspection(s), providing direct access to source data/documents.

Data collected as part of this research included questionnaires, clinical assessments, information from medical records, and qualitative data from interviews. Data was collected through designed questionnaires on paper. These paper forms were scanned at YTU and the data stored in a database where they were checked against the hard copy of the questionnaire. Data was error checked and validation checks were run against the database. Discrepancies identified during validation which required resolution were communicated to the relevant person who was in a position to obtain the information required to rectify the discrepancy. If data were found to be missing from participant completed questionnaires, participants were contacted by one of the research team members in an attempt to collect the data.

DIAMONDS Coaches had the option to record intervention logs electronically via a secure website.

Data management

In line with the 2018 General Data Protection Regulation and the UK Policy Framework for Health and Social Care, anonymised feasibility study data was securely archived by the University of York for a minimum of 10 years. Personal data of participants was stored for up to three years after the study had ended for the purpose of disseminating study findings. It was unlikely that this would take longer than 12 months; however, to ensure that participants received adequate and full information about the study after it had finished, additional time was allocated.

All information collected during the feasibility study was kept strictly confidential as detailed above. Information was held securely in paper and/or electronic formats at the University of York. The University of York complied with all aspects of the 2018 General Data Protection Regulation and Data Protection Act 2018. Operationally, this included obtaining explicit consent from study participants to record personal details including name, postal and email address, and contact telephone numbers, and appropriate storage, restricted access and disposal arrangements for their personal details. All participants were informed of their rights with regard to the personal information stored, including erasure, rectification, and objection. All work was conducted following the University of York’s data protection guidance which was publicly available (University of York, 2018).

A data protection impact assessment (DPIA) was completed and approved by the University of York’s data protection team. In line with University of York policy, this was kept under review throughout the duration of the study.

Confidentiality

At recruitment, each participant was allocated a unique study identification number. This number was used to identify participants throughout the study. The master register linking participants' personal and contact details with the identifier was maintained by the York Trials Unit data manager. Only relevant members of the study team had access to this information via a password-protected database within secure offices. A Participant Screening/Enrolment Log was maintained, providing the dates patients were screened, whether they were eligible or not (with reason), and if consented or not (with reason). This log did not contain any identifiable patient details.

Clinical information was not released without the written permission of the participant, except as necessary for monitoring and auditing by the Sponsor, its designee, Regulatory Authorities, or the REC. The investigator and study site staff involved with this study did not disclose or use for any purpose other than performance of the study, any data, record, or other unpublished, confidential information disclosed to those individuals for the purpose of the study. Prior written agreement from the Sponsor or its designee was obtained for the disclosure of any said confidential information to other parties.

Data security

All data were stored in accordance with data protection requirements and were kept either in a locked filing cabinet in a secure office or in the case of electronic data on a secure server with a password protected computer and files.

Personal addresses, postcodes, and other contact details of consenting participants were stored on a secure password-protected server located at the University of York for the purposes of assisting in follow-ups during the study. All personally identifiable participant data were coded and pseudonymized by participant number in all manual and electronic files. YTU maintained a list of participant identification numbers for all study participants at each site.

Interview recordings were downloaded onto a password-protected computer and deleted from the recording device. They were then securely uploaded to a GDPR-compliant transcribing company.

No data were stored on a home computer or laptop.

All data were stored for a maximum of 10 years, which allowed time for any academic challenge to be made. All personal data were deleted after this time.

# Appendix 3 – Amendments/changes to study protocol

| **Protocol** | **Change from protocol** | **Reasons** |
| --- | --- | --- |
| Participant recruitment from secondary care mental health trusts, primary care, third sector and service user groups. | Participants recruited from secondary care mental health trusts only. | -Time, resource, logistics constraints  -Good progress of recruitment from secondary care |
| Planned 1:1 and group sessions (DIAMONDS intervention) | Group sessions did not take place | COVID 19 restrictions |
| Collection of blood samples for haemoglobin and cholesterol testing | Haemoglobin and cholesterol were not measured | Capacity limits in NHS trusts and the central laboratory due to COVID 19 |
| Collection of health resource use data from primary care records for all participants | Collection of these data was stopped after 6 participants | Capacity limits in primary care and local Clinical Research Network (now Research Delivery Network) due to COVID 19 |

# Appendix 5 – DIAMONDS Coach job description

**Job Description**

| **Job Title** | DIAMONDS Coach |
| --- | --- |
| **Post ref no.** |  |
| **Duration** | Secondment for 24 months |
| **WTE** | 1.0 WTE (part-time possible by negotiation, minimum 0.4 WTE) |
| **Band** | AfC Band 4 |
| **Service area** | Yorkshire & Humber |
| **Location/Base** | Flexible |
| **Accountable to** | Principal Investigator |

1. **Job Purpose:**

**
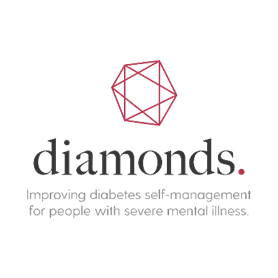
**

This is a unique opportunity for a healthcare practitioner with experience of working within a mental health setting in a service user facing role, and an interest in research to be involved in a multi-centre study. You will gain experience both in a clinical capacity, delivering a healthcare intervention, and in a research capacity, obtaining insight into a large, National Institute for Health and Social Care Research (NIHR) funded research programme. We are looking to recruit healthcare practitioners with recent/current experience of working with adults with serious mental illness and/or type 2 diabetes or with prior experience in delivering behaviour change interventions to become DIAMONDS Coaches and to deliver a diabetes self-management support programme, called DIAMONDS.

You will support people with serious mental illness (schizophrenia, schizoaffective disorder, bipolar disorder, psychosis, severe depression) and type 2 diabetes to achieve new health and wellbeing goals by providing them with one-to-one sessions over a period of six months. Session frequency will vary but is expected to be approximately one session every seven to ten days. The programme will be delivered in a location that is convenient and safe for both you as the Coach and the participant, for example the participant’s home or a local community venue. Sessions may also be delivered by telephone or video call.

As a Coach, you will be responsible for helping the participant to use key components of the DIAMONDS support programme, namely the DIAMONDS workbook and the Change One Thing mobile app, to set and achieve goals. You will need to work both independently and as part of a larger, multi-disciplinary team both within and outside of the Trust.

As a DIAMONDS Coach you will have a unique chance to be part of a large multidisciplinary research project that aims to address important health inequalities, enabling you to broaden your knowledge, skills and experience. You will also undertake online Diabetes UK training, which count towards Continued Professional Development (CPD). Additionally, you will take part in DIAMONDS Coach training, developed by Leicester Diabetes Centre, which will provide you with the skills to deliver the DIAMONDS support programme and a transferable skill set to deliver behaviour change interventions more widely. You will receive ongoing support during your role as a Coach, from within the Trust, the DIAMONDS Research team, and the trainers at Leicester Diabetes Centre.

**Background**

The aim of the DIAMONDS programme is to help people who are living with type 2 diabetes and serious mental illness to improve their diabetes self-management by making small changes to their daily life. The research is funded by the NIHR and we have previously confirmed the acceptability and feasibility of the DIAMONDS support programme. We are now looking to investigate if the intervention changes participants’ blood glucose levels (HbA1c).

People who are over the age of 18 years and have type 2 diabetes and severe mental illness (schizophrenia, schizoaffective disorder, bipolar disorder, psychosis, severe depression) will be recruited to take part in the DIAMONDS trial at sites across the UK.

## Organisational Chart


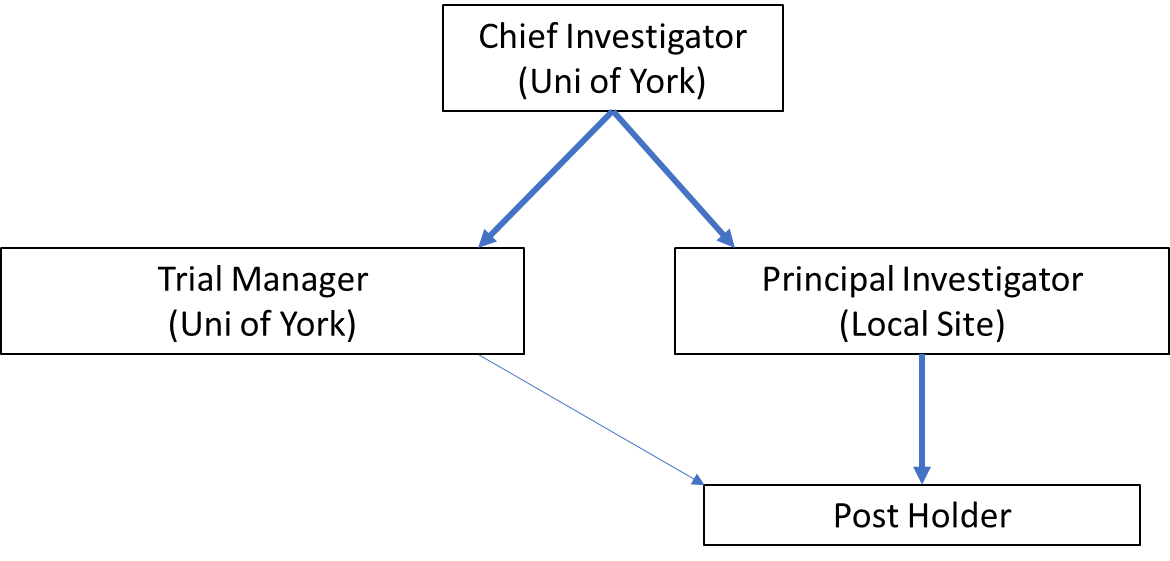


1. **Main duties:**
2. Participate in the DIAMONDS Coach training. This will involve a combination of virtual and in-person workshops as well as self-directed learning for a total of no more than three days. All training materials will be provided.
3. Meet with participants and provide person-centred one-to-one coaching sessions approximately once every seven to ten days to support them to make changes that will improve their health and wellbeing. Depending on WTE, Coaches will work with between 4 (0.4 WTE) and 10 (1 WTE) participants at any one time and will be asked to provide sessions over a six months period. Most sessions will last between 30-60 minutes, with the first and last sessions lasting between 60-90 minutes. These sessions can take place face-to-face or virtually, depending on participant preference and location.
4. Engage participants either in their own homes, in their local community, over telephone or video call, or in local Trust facilities, depending on their preferences. The safety of Coaches and participants is paramount. In-person sessions will only take place if assessed to be safe.
5. Help participants access and use diabetes educational content and be able to provide basic information about diabetes and mental health when needed. Materials will be provided to support you with this.
6. Signpost participants to an appropriate local service or organisation when needs are identified that are outside the scope of the DIAMONDS support programme.
7. Support participants to use the DIAMONDS support programme materials, including the workbook and the Change One Thing app.
8. Produce and maintain up-to-date accurate records relating to one-to-one sessions and report to the DIAMONDS study team in a timely manner. Training on this will be provided.
9. Maintain the privacy and confidentiality of participants and ensure all records and reporting are conducted within general data protection requirements and in line with trust policies.
10. Report all adverse events and participant withdrawals to the DIAMONDS study team in a timely fashion. Training on this will be provided.
11. Respond quickly and appropriately to any safeguarding concerns, in accordance with legal and ethical requirements and study procedures
12. Work within the Trust’s lone working policy. Where none is available, work within the York Trials Unit lone working policy (available on request).
13. Work in a person-centred approach, underpinned by the DIAMONDS support programme philosophy- see below.
14. Behave with honesty and integrity at all times.
15. Liaise with research personnel outside the Trust as necessary.
16. Provide support for colleagues during planned and unplanned absences. Coaches will need to be prepared to work with participants from other Trusts (which may be in other regions). Coaches will be expected to travel locally (within reason) as needed. .
17. Develop and establish good working relationships and maintain high standards of personal and commercial confidentiality with appropriate stakeholders within the Trust and collaborating organisations
18. Deliver care in accordance with study protocol, local policy, Good Clinical Practice Guidance (GCP) and current practice, as applicable.

### Working as part of a Team

1. Maintain effective communication and professional working relationships within the research team, clinical services and other external agencies.
2. Ability to relate to all staff, patients and their carers in a confident manner, demonstrating good listening skills and ability to work effectively as part of the team.
3. Establish and maintain effective communication with participants and their relatives/carers.
4. Contribute your skill and knowledge within the team, both during the DIAMONDS Coach training and while delivering the intervention.
5. Any other duties, which may be requested by the Programme Manager or Principal Investigator, to facilitate the smooth running of the team or study.

### Managing Self

1. Participate in regular supervision.
2. Attend all mandatory training and undertake training to meet the requirements of the role.
3. Comply with all relevant Trust policies, procedures and protocols.
4. To ensure that Trust wide standards are maintained and monitored to improve the quality of care to all those who come in contact with the service provided by the Trust.
5. Carry out duties with due regard to the Trust’s Equal Opportunity Policy.
6. Seek to ensure compliance within the Trust’s policy on data protection, confidentiality and security. To maintain patient confidentiality at all times.
7. Seek advice and support from the Principal Investigator and Programme Manager whenever necessary.
8. To be accountable for own clinical practice, registration and maintain own professional Portfolio as applicable. To keep self-updated and identify own development needs via clinical supervision, training, and appraisal.
9. Treat all individuals with respect.
10. To ensure that the views of service users and carers are effectively sought, channelled and acted upon, including the efficient actioning of the complaints procedure in accordance with Trust policy and reporting procedures for the DIAMONDS research programme.
11. To behave in a manner respecting the differing needs and cultures of other staff and participants.
12. To demonstrate the Trust’s core behaviours in delivering a quality service to both internal and external customers
13. **Staff Supervision and Support**

*No formal staff management or supervisory role.*

1. To challenge any practice that is deemed to be discriminatory or offensive to others.

2. Share latest developments in research and clinical practice.

1. **Financial Responsibility**

1. Ensure safe and efficient use of equipment, resources and consumables at all times.

2. Accurate record keeping of expenses and time spent on travel, session preparation and delivery, record keeping, and training.

3. Maintain appropriate use of IT resources in line with relevant Trust policies.

**8. Safeguarding**

All staff members have a duty to report any concerns they have about the safety or wellbeing of adult service users, or members of their families, including children. Employees should be aware of their roles & responsibilities to both prevent and respond appropriately to abuse. They should undertake the safeguarding training required for their particular role.

**9. Core Values:**

DIAMONDS Support programme philosophy:

- Provide participants with evidence-based, honest information and support them to develop self-management skills while ensuring they have access to the resources, education and support they need. Recognise that participants are ultimately responsible for self-management of their diabetes.
- Ensure all participants you work with are respected and treated non-judgementally regardless of how they manage their condition. Recognise that participants want to maximise their quality of life; understand that quality of life is highly subjective, and your view may differ from the participants’.
- Provide participants with the opportunity and support to reflect on possible barriers to self-management that exist in their own personal and social worlds, and ensure self-management plans account for these barriers.
- Ensure empathy and warmth so participants have a space to explore their experiences of living with severe mental illness and diabetes.

[***To be replaced with trust-specific content as appropriate:***

Below is the Trust’s Vision, Aims and Corporate Priorities. The Corporate Priorities are what the Board has identified as specific priorities for focus for 2014/15.

The Trust’s vision statement is:

**To provide the best possible care for the people of Bradford, Airedale and Craven and to be recognised as one of the country’s leading providers of integrated community health care services**.

Aim 1: To provide a top quality service

Aim 2: To achieve excellence in patient experience

Aim 3: To ensure great relationships between the Trust, its staff and stakeholders

Aim 4: To deliver excellent value for money

**TRUST VALUES**

Respect

Openness

Improvement

Excellence

Together

**Core Statements:**

**1. Infection Control - All clinical and non-clinical staff groups**

Responsible for, in respect to your area of work, for ensuring so far as is reasonably practicable and in accordance with Trust policies that you are aware of your individual responsibilities in regard to infection prevention and control this requires you to:

- Maintain safe infection prevention and control environment for yourself and others.
- Be familiar with and comply with current infection prevention and control, guidelines, policies and procedures.
- Raise matters of non-compliance with your manager.
- Attend infection prevention and control mandatory training as dictated by your manager.
- Be appraised in relation to infection prevention and control.

**2. Risk Management**

All staff need a basic working knowledge of risk management. They all have a responsibility to identify and report risks, hazards, incidents, accidents and near misses promptly, in accordance with Trust Policy. All staff must be familiar with emergency procedures, risk management systems and incident management in their workplace.

**3. Health and Safety**

All employees have a responsibility under the Health and Safety at Work Etc. Act 1974 for their own health, safety and welfare and to ensure that the agreed safety procedures are carried out to provide a safe environment for other employees and anyone else that may be affected by the carrying out of their duties.

Employees must co-operate with the Trust in meeting its statutory obligations with regard to health and safety legislation and must report any accidents, incidents and problems as soon as practicable to their immediate supervisor.

The Trust has a written health and safety policy which employees have a general duty to read in order that they are fully conversant with its requirements.

**4**. **Patient care**

Bradford District Care Trust is committed to ensuring the highest standards of care and treatment and expects that **all** staff employed within the organisation will treat service users, their carers, relatives and friends with dignity and respect at all times during their contact with services we provide.

**5. Information Management**

All members of staff are bound by the requirements of the Data Protection Act 2018 and any breaches of the Act or of the confidential nature of the work of this post could lead to dismissal.

The post holder is responsible to learn about information governance, to help ensure that best practice guidelines are followed and personal information is managed to benefit patients, clients and members of staff.

As part of their usual role the post holder is required to sign the declaration form to confirm they have read and understood the booklet and leaflet regarding information governance, which will be kept by the HR team in the post holder’s personnel file.

| **Job Title** | | DIAMONDS Coach | | |
| --- | --- | --- | --- | --- |
| **Post ref no.** | |  | | |
| **Duration** | | Fixed term for 18 months | | |
| **WTE** | | 1.0 WTE (part-time possible by negotiation, minimum 0.4 WTE) | | |
| **Band** | | AfC Band 4 | | |
| **Service area** | | Yorkshire & Humber | | |
| **Location/Base** | | Flexible | | |
| **Accountable to** | | Principal Investigator | | |
| **Job purpose:**  This is a unique opportunity for a healthcare practitioner with experience in mental health and an interest in research to be involved in a multi-disciplinary study called the DIAMONDS programme. In their role as a DIAMONDS Coach they will support people with serious mental illness and type 2 diabetes to achieve their health and wellbeing goals by providing them with one-to-one sessions over six months. As a Coach, they will be responsible for helping the participant to use the other key components of the DIAMONDS support programme, namely the DIAMONDS workbook and the Change One Thing app, to set an achieve goals. DIAMONDS Coaches will need to work both independently and as part of a larger team. | | | | |
| **Attributes** | **Essential criteria** | | **Desirable criteria** | **How Identified** |
| QUALIFICATIONS | NVQ 3 in Care or equivalent experience | | Postgraduate degree.  Health research qualifications.  Completed GCP training within past 3 years | CV  Interview  Certificates |
| TRAINING | Willingness to undergo any further training to meet requirements of the post, i.e. DIAMONDS Coach training incl. self-directed learning | | Evidence of continuous personal, professional and academic development. | CV  Interview  Expression of interest |
| EXPERIENCE | Proven ability and experience to work effectively autonomously and within a multi-disciplinary team.    Minimum:   - eighteen months experience of working in the NHS in a mental health setting, or - twelve months working in the NHS in a mental health setting with people with serious mental illness.   OR   - twelve months delivering behaviour change, educational, or self-management interventions in an NHS or non-NHS setting | | Experience if working with mental health service users who have type 2 diabetes or another long-term physical health condition  Experience of delivery of service with BME service users, carers and families.  Experience of working to GCP guidelines and/or other regulatory frameworks for clinical research.  Experience of ‘agile working’ and the use of IT for distance working.  Experience of delivery of service evaluation/delivery projects.  Experience of supporting lifestyle change. E.g. smoking cessation  Understanding and experience of research methodology. | CV  Interview |
| KNOWLEDGE | Basic understanding of person-centred practice.  Current professional issues.  Equal Opportunities.  Confidentiality.  Clear knowledge of Health and Safety at Work, and how it relates to this role. | | Knowledge of self-management behaviours and how these apply to type 2 diabetes.  Knowledge of culturally appropriate local supports (statutory and voluntary) available for people with serious mental illness and type 2 diabetes | CV  Interview |
| SKILLS | Excellent written & verbal communication skills to include:   - Able to liaise with, and explain concepts to, people with serious mental illness. - Able to communicate difficult matters in challenging situations.   Ability to negotiate and work collaboratively with service users, Trust staff, and the research team.  Must be able to work effectively under pressure and to respond appropriately to peaks of work activities, by prioritising and organising own work with minimal supervision.  Able to organise working with multiple participants simultaneously within the intervention schedule.  Able to work flexibly in order to work round participant’s commitments and needs.  Good Computer skills to include: -   - Word Processing and use of Excel spread sheets - Use of Outlook/email - Use of video conferencing software, such as Zoom/MS Teams. - Use of electronic patient records eg. SystmOne - Ability to access training resources and treatment logs online   Proven ability to problem-solve  Ability to work independently and on own initiative.  Ability to travel independently to various locations. | |  | CV  Interview |
| ATTITUDE/APPROACH | Committed to teamwork: valuing and supporting others to achieve their goals.  Capable of developing and sustaining good working relationships with colleagues, the study team, and participants.  Positive, non-judgemental, and enthusiastic disposition.  Treats people with respect and understanding regardless of cultural educational background and experience.  A committed approach to attendance and takes responsibility fulfilling the requirements of the role.  Creative and adaptable.  Desire and willing to learn and share new skills and knowledge. | |  | CV  Interview |
| PHYSICAL | Able to fulfil Occupational Health requirements for the post (with reasonable adjustments if necessary). | |  | Occupational Health Screening |
| GENERAL | **BDCFT requires all its staff and prospective employees to carry out your duties in line with Trust Equality policies and procedures, including relevant legislation, to deliver and promote equity of access to healthcare and equality of opportunity at work at all times.** | | Access to a car and current valid driver’s license | CV  Interview |

# Appendix 6 – DIAMONDS intervention specification

Please note that this is the specification for the first iteration of the DIAMONDS intervention that was tested in the feasibility study reported here. The intervention was revised and refined iteratively in response to findings from the feasibility study.


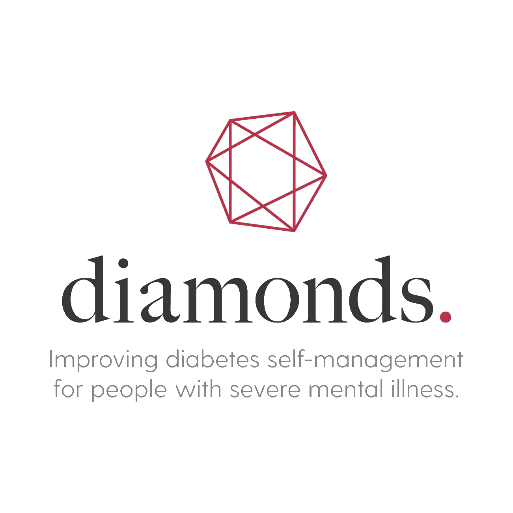

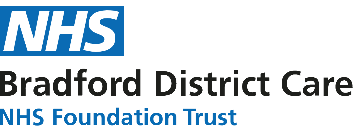

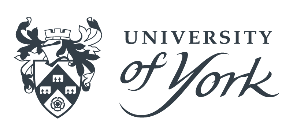

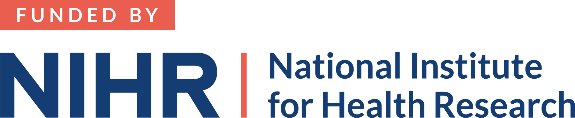


**INTERVENTION SPECIFICATION**

**A tailored intervention to support self-management of Type 2 diabetes in people with severe mental illness (SMI)**

This evidence-based behavioural intervention aims to improve diabetes self-management in adults with severe mental illness (SMI) and Type 2 diabetes. The co-designed intervention supports self-management through:

- increasing knowledge and skills for diabetes self-management
- supporting participants to increase physical activity levels and eat healthier
- identifying and addressing barriers to taking medications
- identifying and addressing sleep problems
- helping participants to manage diabetes within the context of fluctuating and low mood
- facilitating peer support.

The intervention will be delivered using a combination of behaviour change techniques (BCTs) (e.g. goal setting, self-monitoring) that offer the most promise to modify the individual and environmental processes that influence self-management behaviours in this patient group (e.g. motivation, intentions, emotion). We refer to these processes as ‘mechanisms of action’ (MOAs), and have prioritised 15 MOAs which we will target in the intervention (see Table 1).

The BCTs (which are the active ingredients of an intervention) were selected according to the evidence linking them to targeted MOAs, their acceptability to people with SMI, and the feasibility of delivering them as part of a self-management intervention within existing healthcare services (see Table 2).

The selected BCTs will be delivered by a trained facilitator (‘the DIAMONDS Coach’), over 16 weeks, using a combination of individual weekly sessions and daily use of a digital app and paper-based workbook, and monthly group sessions (see Figure 1 and details below).

**Figure 1 – The Diamonds Intervention**

**Weekly sessions**

All participants will receive 16 individual weekly sessions with the DIAMONDS Coach (in a venue chosen by the participant, e.g. their home, an NHS facility, a café). The first session will last between 60 and 90 minutes and follow-up sessions will each last between 30 and 60 minutes depending on the needs and preferences of the participant. All sessions will ideally be delivered in person, however follow-up sessions may be delivered by phone or video call if participants express a preference for this.

The aim of the weekly sessions is to provide information about diabetes, support participants to increase physical activity levels and make other healthy lifestyle changes, and set goals and implement plans to improve sleep or medication taking. The DIAMONDS coach will use the digital app (Change One Thing) and workbook to deliver a combination of BCTs. As part of their role they will support the participant to set-up the app and prepare the workbook in the first weekly session, and provide guidance about how to use these resources between sessions.

**Digital app (Change One Thing)**

Change One Thing is a digital app for use on Android and IoS devices. It requires an Internet connection to enable full functionality, and has been co-designed by service users and carers. All participants will be encouraged to use the app as part of the intervention, and will receive support and encouragement for this from the DIAMONDS coach during weekly sessions.

Change One Thing aims to support participants to set goals with the DIAMONDS coach, and to achieve the goals set by delivering daily prompts, making physical activity recommendations based on participant preference, mood and the weather, providing information about the consequences of self-management, and enabling self-monitoring.

**Workbook**

The paper-based workbook will be used by the DIAMONDS coach and participant together in the weekly sessions, and by the participant as needed in between sessions. The workbook is divided into four sections:

1. information about diabetes and diabetes self-management, delivered in discrete topics (e.g. What is diabetes?, checking blood sugar, healthy eating, getting active, taking medication, preventing complications, getting help). This section will also contain a checklist to assess information needs in the first weekly session and record information provision over the 16 weeks.
2. A physical activity goal setting record sheet for the DIAMONDs coach and participant to use each week.
3. a paper-based version of the Change One Thing app so that participants who do not wish to or who are not able to use the app are still able to access these elements of the intervention.
4. personalised information about who the participant should contact if they experience problems with their mental health or diabetes, who in their social network can provide social support, and details of local services available to them to support diabetes self-management (e.g. peer support groups, exercise classes). This will be completed by the DIAMONDS coach with the participant in the first weekly session.

In combination, the weekly sessions, app and workbook will deliver the following BCTs:

***Goal setting and action planning***
In week 1, the DIAMONDS coach will support the participant to set goals for improving sleep or medication-taking in Change One Thing (app or paper-based version) and make action plans for these. The coach will also support the participant to select activities that they would like to try during the following week and enter these into Change One Thing. The coach and participant will set a physical activity goal based on these preferences. This will be recorded in the workbook.

***Graded tasks***
The DIAMONDS coach will help the participant to set physical activity goals that are easy to perform initially, and encourage them to set more difficult goals over the 16 weeks.

***Problem-solving***
In all weekly sessions, the coach will identify participant barriers to engaging in self-management and accessing support for this, and use problem-solving techniques to help participants address these.

***Focus on past success***
In all weekly sessions, participants will be encouraged to focus on their past successes in managing their diabetes. Physical activity-related successes that have helped participants to manage their mood will be entered into Change One Thing so that participants can focus on these in between sessions.

***Prompts / cues***
In week 1, the DIAMONDS coach will help participant to set prompts for each action plan (typically times of regular daily events, such as waking up, leaving the house, lunchtime, bedtime). These will be entered into Change One Thing to trigger daily smartphone reminders (alarms & visual notifications with text), and written in the workbook. For participants who do not want to use the app, the coach will work with the participant to create prompts in the person’s own environment.

Change One Thing will also prompt participants to engage in daily physical activity, with suggestions tailored to participant preference (activities selected with the coach in week 1), mood (whether participants are having a good day or a bad day) and the weather (indoor and outdoor activities).

The prompts will be revised each week as goals and action plans change.

***Reduce negative emotions***
The DIAMONDS coach will advise participants to engage in physical activity and practice relaxation techniques to reduce negative emotions. Change One Thing will select activities based on participant mood, with lower intensity activities suggested on days that participants are finding difficult.

***Self-monitoring of behaviour and Self-monitoring of outcome of behaviour***
Participants will be prompted to self-monitor their progress with action plans for sleep or medication daily in Change One Thing. Participants will also be asked to record their physical activity and the impact of this on their mood. In Change One Thing this will be based on activities they select to try daily. In the workbook, this will relate to the goal set with the coach each week. Self-monitoring records in Change One Thing will be visible for use by participants and by the coach and participant in weekly sessions.

***Non-specific rewards***
When participants record their daily activities in Change One Thing, a ‘reward’ statement will be generated depending on the response.

***Feedback on behaviour, Feedback on outcome of behaviour, and Review behaviour goals***
The self-monitoring record will be reviewed in weekly sessions 2-16 with the DIAMONDS coach, who will provide feedback on the behaviour change achieved, and the outcome of these behaviours, e.g. improvements in sleep, improvements in mood. The DIAMONDS coach will then repeat the process of guiding the participants through the goal setting and action planning aspects described above, enabling the participants to continue with or revise an existing goal or set a new goal, and revise the action plans as required.

***Monitoring of emotional consequences***In each weekly session, the DIAMONDS coach will ask the participant to reflect on the emotional consequences of any changes in their behaviour. The self-monitoring record will be used as part of this discussion. Problem-solving techniques may also be used here if negative consequences are identified.

***Information about health consequences and Information about emotional consequences***
In the first week, the DIAMONDS coach will assess the information needs of the participant using a checklist of diabetes topics. Each week the participant and coach will decide together which topic to focus on, and the DIAMONDS coach will work through the relevant section of the workbook with the participant, with content focused around the consequences of diabetes self-management. Each section will contain signposting for participants if they wish to access further information.

Change One Thing also delivers short animations about the health and emotional consequences of improving sleep, medication-taking and increasing physical activity.

***Instruction on how to perform the behaviour and Demonstration of the behaviour***The DIAMONDS coach will provide instructions for self-management behaviours in weekly sessions where these are needed, and will demonstrate behaviours (e.g. go for walk with the participant) or help participants to access demonstrations (e.g. through YouTube videos) where needed. The workbook will also contain instructions for core self-management activities (e.g. interpreting blood sugar readings, where needed, healthy eating tips, checking feet).

***Verbal persuasion***
The Diamonds coach will use verbal persuasion techniques to address any doubts participants have about their capabilities to make lifestyle changes and manage their diabetes.

***Behavioural practice / rehearsal and Habit formation***
Change One Thing encourages daily practice and habit formation of sleep hygiene / medication taking, and physical activity. The Diamonds coach will reinforce this in weekly sessions.

***Social support (practical) and Social support (emotional)***
In the first week, the DIAMONDS coach will identify sources of social support available to the participant and encourage them to utilise these. They will also provide details of local services available to participants to support diabetes self-management. These will all be recorded in the workbook. In subsequent sessions, participants will be asked to reflect on any support they have received / accessed. The coach will use problem-solving techniques to help participants address barriers to accessing support.

**Monthly sessions**

Monthly group sessions will be provided by two trained facilitators (in a community-based venue, e.g. local town hall, community hub). Sessions will be held in the afternoons for 90 minutes. A healthy lunch will be provided at the start of each group session. Sessions will be attended by 6-10 participants who are receiving the intervention.

The aim of the sessions is to facilitate peer support among people with SMI and diabetes. Each session will follow the same format and use the following BCTs:

***Demonstration of behaviour*** – a healthy lunch will be provided at each session

***Focus on past success*** – participants will be asked to focus on and share their successes in managing their diabetes in each session

***Problem-solving*** – in each session participants will be asked to talk about the challenges (e.g. triggers for poor self-management, barriers to engaging in self-management behaviours, barriers to using the digital app) they have experienced so far and identify solutions with the facilitators and other participants.

***Social support (unspecified)*** – participants will be encouraged to offer each other encouragement and support throughout the sessions.

**Table 1 – Mechanisms of Action (MOAs) targeted by the DIAMONDS intervention**

| Mechanism of Action (MOA) | Example quote from DIAMONDS Quest study |
| --- | --- |
| **Knowledge**: An awareness of the existence of something | *I always take my medication because I know I need it, I never miss it.* |
| **Skills**: An ability or proficiency acquired through practice | *I wasn’t quite using my inhalers and this COPD Nurse told me to use it better. And she advised me to use it properly. I was taking it but not fully using it correctly.* |
| **Beliefs about capabilities**: Beliefs about one’s ability to successfully carry out a behaviour | *I can’t be active because I’ve got mobility issues.* |
| **Beliefs about consequences**: Beliefs about the consequences of a behaviour (i.e. perceptions about what will be achieved and or lost by undertaking a behaviour, as well as the probability that a behaviour will lead to a specific outcome) | *Smoking stops me from getting too anxious, my nerves and all, it just helps me relax and that, it does help me a lot.* |
| **Reinforcement**: Processes by which the frequency or probability of a response is increased through a dependent relationship or contingency with a stimulus or circumstance | *I’ve joined the gym and that’s helping a lot because exercise not only helps the body, it helps the mind.* |
| **Intentions**: A conscious decision to perform a behaviour or a resolve to act in a certain way. | *I always do take my meds and I get them ready before I go to bed.* |
| **Goals**: Mental representations of outcomes or end states that an individual wants to achieve | *I’d like to lose more weight, get down to maybe nine stone. I’m 12 stone at the minute.* |
| **Memory, attention and decision processes**: Ability to retain information, focus on aspects of the environment and choose between two or more alternatives | *I’m terrible at remembering to go for it but I think I’m supposed to have a blood test every few months to check the levels.* |
| **Environmental context and resources**: Aspects of a person’s situation or environment that discourage or encourage the behaviour | *I don’t walk much, I walk to the gym, I don’t like walking in the neighbourhood, I think there’s too many cars and houses, there’s not enough greenery.* |
| **Emotion**: A complex reaction pattern involving experiential, behavioural and physiological elements | *It makes it sort of hard even just to go to the supermarket or you know, anywhere. So, it does, like, depression wise, it does get to the point where I just can’t function.* |
| **Behavioural regulation**: Behavioural, cognitive and/or emotional skills for managing or changing behaviour | *I have a pill box and I just take it without fail in the morning and at night time.* |
| **Attitude towards the behaviour**: The general evaluations of the behaviour on a scale ranging from negative to positive | *I have an injection every fortnight and that keeps me stable. I don’t like it, it hurts, it’s horrible but it keeps me well.* |
| **Motivation**: Processes relating to the impetus that gives purpose or direction to behaviour and operates at a conscious or unconscious level | *I feel more motivated because it’s an incentive that I’ve lost weight and it’s like lifted my mood as well.* |
| **Feedback processes**: Processes through which current behaviour is compared against a particular standard | *Sometimes when I have bad results of weigh-ins that puts me off and it makes me wonder shall I even bother continuing.* |
| **Behavioural cueing**: Processes by which behaviour is triggered from either the external environment, the performance of another behaviour or from ideas appearing in consciousness | *Appointments, I manage to attend them because I get texts on my phone from the doctors.* |

**Table 2 – Behaviour Change Techniques (BCTs) which comprise the DIAMONDS intervention**

**THIS NEEDS RE-MAPPING AND UPDATING AFTER DESIGN IS FINALISED**

| Behaviour Change Technique (BCT) | Linked Mechanism(s) of Action |
| --- | --- |
| **Goal setting (behaviour)**: Set or agree on a goal defined in terms of the behaviour to be achieved | Intentions; Goals; Memory, attention, and decision processes |
| **Problem solving**: Analyse or prompt the person to analyse, factors influencing the behaviour and generate or select strategies that include overcoming barriers and/or increasing facilitators | Knowledge; Skills; Beliefs about capabilities; Memory, attention, and decision processes; Behavioural regulation |
| **Action planning**: Prompt detailed planning of performance of the behaviour (must include at least one of context, frequency, duration and intensity). Context may be environmental (physical or social) or internal (physical, emotional or cognitive) | Memory, attention, and decision processes; Behavioural regulation; Behavioural cueing |
| **Review behaviour goal(s)**: Review behaviour goal(s) jointly with the person and consider modifying goal(s) or behaviour change strategy in light of achievement. This may lead to re-setting the same goal, a small change in that goal or setting a new goal instead of (or in addition to) the first, or no change | Beliefs about capabilities; Reinforcement; Intentions; Goals; Feedback processes |
| **Feedback on behaviour**: Monitor and provide informative or evaluative feedback on performance of the behaviour (e.g. form, frequency, duration, intensity) | Reinforcement; Motivation; Feedback processes |
| **Self-monitoring of behaviour**: Establish a method for the person to monitor and record their behaviour(s) as part of a behaviour change strategy | Behavioural regulation; Feedback processes; Behavioural cueing |
| **Self-monitoring of outcome(s) of behaviour**: Establish a method for the person to monitor and record the outcome(s) of their behaviour as part of a behaviour change strategy | Goals |
| **Feedback on outcome(s) of behaviour**: Monitor and provide feedback on the outcome of performance of the behaviour | Reinforcement; Goals; Feedback processes |
| **Social support (unspecified)**: Advise on, arrange or provide social support (e.g. from friends, relatives, colleagues,’ buddies’ or staff) or noncontingent praise or reward for performance of the behaviour. It includes encouragement and counselling, but only when it is directed at the behaviour | Environmental context and resources |
| **Social support (practical)**: Advise on, arrange, or provide practical help (e.g. from friends, relatives, colleagues, ‘buddies’ or staff) for performance of the behaviour | Environmental context and resources |
| **Social support (emotional)**: Advise on, arrange, or provide emotional social support (e.g. from friends, relatives, colleagues, ‘buddies’ or staff) for performance of the behaviour | Emotion |
| **Instruction on how to perform the behaviour**: Advise or agree on how to perform the behaviour | Knowledge; Skills; Beliefs about capabilities |
| **Information about health consequences**: Provide information (e.g. written, verbal, visual) about health consequences of performing the behaviour | Knowledge; Beliefs about consequences; Intentions; Attitude towards behaviour |
| **Monitoring of emotional consequences**: Prompt assessment of feelings after attempts at performing the behaviour | Beliefs about consequences; Emotion |
| **Information about emotional consequences**: Provide information (e.g. written, verbal, visual) about emotional consequences of performing the behaviour | Knowledge, Beliefs about consequences; Emotion |
| **Demonstration of the behaviour**: Provide an observable sample of the performance of the behaviour, directly in person or indirectly e.g. via film, pictures, for the person to aspire to or imitate | Knowledge; Skills; Beliefs about capabilities |
| **Prompts/cues**: Introduce or define environmental or social stimulus with the purpose of prompting or cueing the behaviour. The prompt or cue would normally occur at the time or place of performance | Memory, attention, and decision processes; Environmental context and resources; Behavioural regulation; Behavioural cueing |
| **Behavioural practice/rehearsal**: Prompt practice or rehearsal of the performance of the behaviour one or more times in a context or at a time when the performance may not be necessary, in order to increase habit and skill | Skills; Beliefs about capabilities; Behavioural regulation |
| **Habit formation**: Prompt rehearsal and repetition of the behaviour in the same context repeatedly so that the context elicits the behaviour | Memory, attention, and decision processes; Behavioural regulation; Behavioural cueing |
| **Graded tasks**: Set easy-to-perform tasks, making them increasingly difficult, but achievable, until behaviour is performed | Skills; Beliefs about capabilities; Memory, attention, and decision processes |
| **Reduce negative emotions**: Advise on ways of reducing negative emotions to facilitate performance of the behaviour | Skills; Emotion; Behavioural regulation |
| **Conserving mental resources**: Advise on ways of minimising demands on mental resources to facilitate behaviour change | Memory, attention, and decision processes; Behavioural regulation |
| **Verbal persuasion about capability**: Tell the person that they can successfully perform the wanted behaviour, arguing against self-doubts and asserting that they can and will succeed | Beliefs about capabilities |
| **Focus on past success**: Advise to think about or list previous successes in performing the behaviour (or parts of it) | Beliefs about capabilities |

# Appendix 7 – Additional outcomes tables

**Appendix S7: Outcome data tables**

**Table S1. Summary of diabetes specific outcomes**

|  | **Participants analysed (n=29)** |
| --- | --- |
| **HbA1c (mmol/mol)**  n (%)  Mean (SD) | 22 (75.9%)  64.2 (14.3) |
| **Prescribed insulin to treat diabetes, n (%)**  *Number with data*  Yes  No | *28 (96.6%)*  7 (24.1%)  21 (72.4%) |
| **PAID-5** | |
| **PAID-5 score (range 0-20)**  n (%)  Mean (SD) | 29 (100%)  5.8 (5.5) |
| **Participants in high distress, n (%)**  *Number with data*  Not in high distress (PAID score <8)  High distress (PAID score ≥8) | *29 (100%)*  19 (65.5%)  10 (34.5%) |
| **SDSCA (scores represent number of days 0-7)** | |
| **General Diet Score (days)**  n (%)  Mean (SD)  Median (IQR) | 28 (96.6%)  2.8 (2.7)  3.3 (0.0-5.0) |
| **Specific Diet Score (days)**  n (%)  Mean (SD)  Median (IQR) | 28 (96.6%)  3.1 (1.7)  3.5 (2.5-4.0) |
| **Exercise Score (days)**  n (%)  Mean (SD)  Median (IQR) | 29 (100%)  2.1 (2.4)  1.5 (0.0-3.5) |
| **Blood-Glucose Testing Score (days)**  n (%)  Mean (SD)  Median (IQR) | 27 (93.1%)  2.2 (2.7)  1.0 (0.0-3.5) |
| **Foot Care Score (days)**  n (%)  Mean (SD)  Median (IQR) | 29 (100%)  2.0 (2.4)  0.5 (0.0-3.5) |
| **Smoked in the last 7 days, n (%)**  *Number with data*  Yes  No | *29 (100%)*  7 (24.1%)  22 (75.9%) |
| **Number of cigarettes smoked on an average day**  n (% of smokers)  Mean (SD) | 7 (100%)  20.4 (14.6) |

**Table S2. Summary of mental health specific outcomes**

|  | Participants analysed (n=29) |
| --- | --- |
| **BPRS** | |
| **BPRS total score (range 18-126)**  n (%)  Mean (SD) | 29 (100%)  34.2 (11.7) |
| **BPRS total score Group, n (%)**  *Number with data*  No symptoms (≤ 24)  Mildly ill (25-32)  Moderately ill (33-49)  Markedly ill (50-69)  Severely ill (70-89)  Extremely ill (≥90) | *29 (100%)*  6 (20.7%)  7 (24.1%)  14 (48.3%)  1 (3.4%)  1 (3.4%)  0 (0.0%) |
| **PHQ-9** | |
| **PHQ-9 score (range 0-27)**  n (%)  Mean (SD) | 29 (100%)  12.8 (6.5) |
| **PHQ-9 Score Group, n (%)**  *Number with data*  No Depression (≤4)  Mild Depression (5-9)  Moderate Depression (10-14)  Moderately Severe Depression (15-19)  Severe Depression (20-27) | *29 (100%)*  6 (20.7%)  2 (6.9%)  9 (31.0%)  8 (27.6%)  4 (13.8%) |
| **How difficult have these problems made it for you to do your work, things at home, or get along with other people, n (%)**  *Number with data*  Not difficult at all  Somewhat difficult  Very difficult  Extremely difficult | *29 (100%)*  8 (27.6%)  9 (31.0%)  7 (24.1%)  5 (17.2%) |

**Table S3: Summary of physical activity measured by the International Physical Activity Questionnaire (IPAQ).**

|  | **Participants analysed**  **(n=29)** |
| --- | --- |
| ***IPAQ*** | |
| **Low physical activity (mins/week)***  n (%)  Mean (SD)  Median (IQR)  Min, Max | 25 (86.2%)  74.8 (135.4)  0.0 (0.0-120.0)  (0.0, 420.0) |
| **Moderate physical activity (mins/week)**  n (%)  Mean (SD)  Median (IQR)  Min, Max | 29 (100%)  44.5 (158.5)  0.0 (0.0-0.0)  (0.0, 840.0) |
| **Vigorous physical activity (mins/week)**  n (%)  Mean (SD)  Median (IQR)  Min, Max | 29 (100%)  46.6 (119.9)  0.0 (0.0-0.0)  (0.0, 480.0) |
| **Total physical activity (MET-mins/week)***  n (%)  Mean (SD)  Median (IQR)  Min, Max | 29 (100%)  763.1 (1722.9)  0.0 (0.0-720.0)  (0.0, 8106.0) |
| **IPAQ score categories, n (%)***  *Number with data*  Low  Moderate  High | *29 (100%)*  24 (82.8%)  4 (13.8%)  1 (3.4%) |
| **Sedentary activity (mins/weekday)**  n (%)  Mean (SD)  Median (IQR)  Min, Max | 27 (93.1%)  551.5 (325.0)  540.0 (300.0-720.0)  (10.0, 1440.0) |

SD, Standard deviation. IQR, Interquartile range.

*Due to an error in the question, time spent doing low activity may be incorrect, consequently, total activity may be underestimated.

**Table S4: Summary of health-related quality of life EQ-5D-5L questionnaire.**

|  | **Participants analysed**  **(n=29)** |
| --- | --- |
| **EQ-5D-5L Mobility domain, n (%)**  *Number with data*  No problems  Slight problems  Moderate problems  Severe problems  Extreme problems/unable to do | *29 (100%)*  13 (44.8%)  2 (6.9%)  7 (24.1%)  7 (24.1%)  0 (0.0%) |
| **EQ-5D-5L Self-care domain, n (%)**  *Number with data*  No problems  Slight problems  Moderate problems  Severe problems  Extreme problems/unable to do | *29 (100%)*  18 (62.1%)  5 (17.2%)  5 (17.2%)  1 (3.4%)  0 (0.0%) |
| **EQ-5D-5L Usual Activities, n (%)**  *Number with data*  No problems  Slight problems  Moderate problems  Severe problems  Extreme problems/unable to do | *28 (96.6%)*  12 (42.9%)  7 (25.0%)  5 (17.9%)  4 (14.3%)  0 (0.0%) |
| **EQ-5D-5L Pain/Discomfort domain, n (%)**  *Number with data*  No problems  Slight problems  Moderate problems  Severe problems  Extreme problems/unable to do | *29 (100%)*  13 (44.8%)  5 (17.2%)  5 (17.2%)  5 (17.2%)  1 (3.4%) |
| **EQ-5D-5L Anxiety/Depression domain, n (%)**  *Number with data*  No problems  Slight problems  Moderate problems  Severe problems  Extreme problems/unable to do | *29 (100%)*  8 (27.6%)  9 (31.0%)  5 (17.2%)  5 (17.2%)  2 (6.9%) |
| **EQ-5D-5L VAS score**  n (%)  Mean (SD) | 29 (100%)  62.9 (25.3) |
